# Interneuron hypomyelination is associated with cognitive inflexibility in a rat model of schizophrenia

**DOI:** 10.1038/s41467-020-16218-4

**Published:** 2020-05-11

**Authors:** Dorien A. Maas, Vivian D. Eijsink, Marcia Spoelder, Josephus A. van Hulten, Peter De Weerd, Judith R. Homberg, Astrid Vallès, Brahim Nait-Oumesmar, Gerard J. M. Martens

**Affiliations:** 1 Department of Molecular Animal Physiology, Donders Institute for Brain, Cognition and Behavior, Centre for Neuroscience, Faculty of Science, Radboud University, Geert Grooteplein Zuid 26-28, 6525 GA Nijmegen, The Netherlands; 2 Paris Brain Institute, ICM, Inserm U1127, Sorbonne Université, CNRS UMR 7225, Hôpital Pitié-Salpêtrière, Paris, France; 3 Department of Cognitive Neuroscience, Donders Centre for Medical Neuroscience, Radboud University Medical Center, Geert Grooteplein 21, 6525 EZ Nijmegen, The Netherlands; 4 Department of Neurocognition, Faculty of Psychology and Neurosciences, Maastricht University, 6200 MD Maastricht, The Netherlands; 5 Maastricht Centre of Systems Biology (MaCSBio), Faculty of Science and Engineering, Maastricht University, 6200 MD Maastricht, The Netherlands

**Keywords:** Schizophrenia, Oligodendrocyte

## Abstract

Impaired cognitive functioning is a core feature of schizophrenia, and is hypothesized to be due to myelination as well as interneuron defects during adolescent prefrontal cortex (PFC) development. Here we report that in the apomorphine-susceptible (APO-SUS) rat model, which has schizophrenia-like features, a myelination defect occurred specifically in parvalbumin interneurons. The adult rats displayed medial PFC (mPFC)-dependent cognitive inflexibility, and a reduced number of mature oligodendrocytes and myelinated parvalbumin inhibitory axons in the mPFC. In the developing mPFC, we observed decreased myelin-related gene expression that persisted into adulthood. Environmental enrichment applied during adolescence restored parvalbumin interneuron hypomyelination as well as cognitive inflexibility. Collectively, these findings highlight that impairment of parvalbumin interneuron myelination is related to schizophrenia-relevant cognitive deficits.

## Abstract (web summary)

Dysfunction of GABAergic neurons in the prefrontal cortex has been reported in schizophrenia. Here, the authors use the apomorphine-susceptible rat, which displays some schizophrenia-like behaviors, and show that interneurons in the medial prefrontal cortex are hypomyelinated, which may contribute to this behavioral phenotype.

## Introduction

Higher cognitive functions, such as working memory and attentional flexibility develop during adolescence, are optimal in early adulthood and are dependent on correct maturation of the prefrontal cortex (PFC)^1^. Interneurons in the PFC microcircuit as well as proper PFC myelination are known to be crucial for proper cognitive functioning^2,3^. Cognitive impairment is a core feature of schizophrenia (SZ), a complex neuropsychiatric disorder affecting 1% of the world population and with a large economic burden^4^. Human genetic and postmortem, as well as animal model studies have suggested an intrinsic defect of interneurons in SZ (refs. ^5–10^). GABAergic interneurons play an important regulatory role in cortical microcircuits and encompass ~20% of all myelinated axons in the neocortex^11,12^. The interneuron defect in SZ is characterized by a reduced PFC expression of GABA-related mRNAs, while the number and dendritic arborization of PFC interneurons is not affected^13–15^. In addition, in the PFC of SZ patients lower myelin levels are found^16^ and postmortem SZ brain tissue shows reduced myelin- and oligodendrocyte (OL)-related gene expression levels^17,18^. Rodent models of SZ are also characterized by myelination defects^19,20^, whereas conversely animal models of demyelination display SZ-related behavioral characteristics^21^. It is further noteworthy that the onset of SZ coincides with both interneuron maturation and PFC myelination during late adolescence. On the basis of these findings, defective myelination of PFC interneurons during adolescence has been hypothesized to underlie cognitive impairment in SZ (refs. ^14,22^). However, no studies have assessed the development and characteristics of PFC interneuron myelination in this disorder^14^.

Understanding the causal pathophysiology of SZ will allow the development of new treatment strategies. However, because of its highly complex nature SZ is mechanistically enigmatic and not easy to mimic in animals. Yet, animal studies are pivotal for mechanistic insight. A number of animal models exist, in which a combination of genetic and environmental factors generates a SZ-related phenotype^23^. One such model is the apomorphine-susceptible (APO-SUS) rat that, together with its phenotypic counterpart the apomorphine-unsusceptible (APO-UNSUS) rat, has been extensively studied as an idiopathic model of SZ (refs. ^24,25^). Without requiring genetic or pharmacological manipulation, APO-SUS rats show SZ-relevant behavioral traits in the positive (e.g., reduced prepulse inhibition, increased exploratory behavior, and dopamine-induced stereotypic behavior)^26–28^, negative (e.g., reduced latent inhibition and sucrose preference)^29,30^, and cognitive (e.g., memory deficit)^31^ domains, as well as neurobiological similarities with SZ patients, such as a hyperactive HPA axis and elevated dopamine D2-receptor binding^15,32^. In particular, APO-SUS rats have a defective PFC microcircuit due to a reduced excitability of GABAergic interneurons, reminiscent of the interneuron abnormalities described in SZ patients^15^. We use the APO-SUS and APO-UNSUS rats as a model to examine the role of (developmental) interneuron myelination in PFC-dependent cognitive impairment. Here, we report that APO-SUS rats display cognitive inflexibility, that interneurons are hypomyelinated during PFC development, and that environmental enrichment during adolescence restores interneuron hypomyelination and rescues cognitive impairment.

## Results

### mPFC-specific cognitive inflexibility in APO-SUS rats

**Fig. 1:**
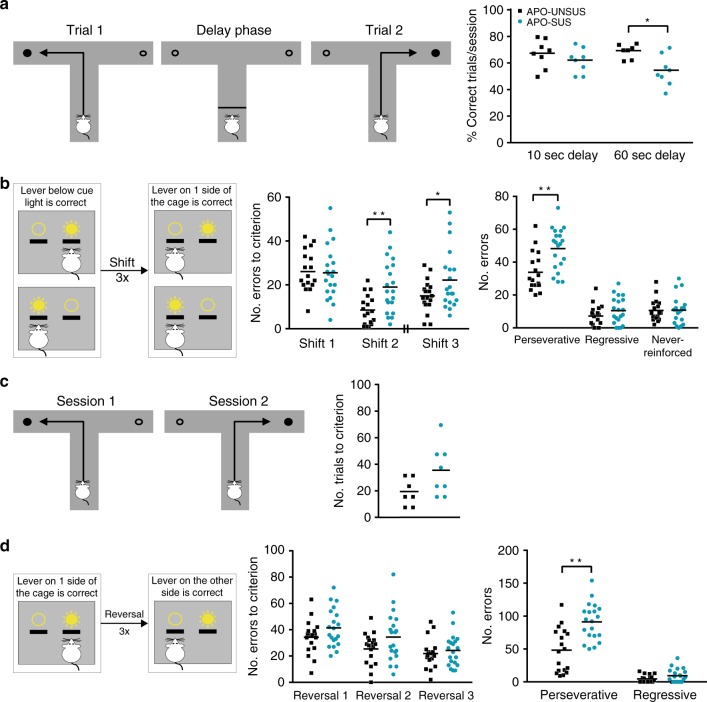
mPFC-dependent cognitive flexibility, but not reversal learning, is impaired in APO-SUS versus APO-UNSUS rats. **a** Schematic representation of the continuous delayed alternation test. Rats were required to retrieve a food reward from alternating arms of a T-maze. Delay periods were administered in separate sessions. Average percentage (%) of correct trials per session in APO-UNSUS (10-s delay *n* = 8; 60-s delay *n* = 7) and APO-SUS rats (*n* = 8) is depicted; *p = 0.015 in two-tailed independent samples *t*-test**. b** Schematic representation of the extra-dimensional set-shifting task. Number of errors to criterion (streak of ten correct trials) and total number of perseverative (following the ‘old rule’), regressive (following the ‘old rule’ while >70% of previous trials were correct), or never-reinforced (pressing a lever that was incorrect during both the ‘old rule’ and during the current rule) errors in APO-UNSUS (*n* = 18) and APO-SUS rats (*n* = 20) are depicted. Note that between shift 2 and shift 3 the reversal learning trials were conducted (see **d**). Shift 2 **p = 0.002, shift 3 *p = 0.048, perseverative errors *p = 0.001 in two-tailed independent samples *t*-test. **c** Schematic representation of T-maze reversal learning paradigm. Number of trials until criterion of 70% correct performance in APO-UNSUS (*n* = 7) and APO-SUS rats (*n* = 8) is depicted. **d** Schematic representation of operant reversal learning paradigm. Number of errors until criterion of a streak of ten correct trials, and total number of perseverative and regressive errors in APO-UNSUS (*n* = 18) and APO-SUS rats (*n* = 20) is depicted; **p < 0.0001 in two-tailed independent samples *t*-test. Source data are provided as a Source data file.

To explore cognitive functioning of the APO-SUS rats relative to that of the APO-UNSUS rats, we performed SZ-relevant cognitive behavioral tests in adulthood (postnatal day (P)90). The first test was to examine spatial working memory using the continuous delayed alternation paradigm with 10-s or 60-s delays between trials (each delay was conducted in five separate sessions; Fig. 1a). A trial was counted correct when the first arm entry was into the baited arm and performance was quantified as the percentage correct trials per session. Independent samples *t*-tests showed no difference in performance per session at the 10-s delay (*t* = −1.043, *p* = 0.315, df = 14), but confirmed a significant decrease in the 60-s delay performance per session (*t* = −2.947, *p* = 0.015, df = 9.858) in APO-SUS versus APO-UNSUS rats. These findings indicate that mPFC-linked spatial working memory is impaired in APO-SUS rats.

To confirm mPFC dysfunction, we next performed an extra-dimensional operant set-shifting test^33^. During this test rats were trained to press the lever above which a cue light was illuminated (visual cue discrimination) until they reached a criterion of ten correct trials in a row. There were no differences in the number of errors made during visual cue discrimination in APO-SUS and APO-UNSUS rats (Supplementary Fig. 1). During the next session, the extra-dimensional shift was introduced, and during that a reward was provided when the animal pressed the lever on one side of the cage (the non-preferred lever, as revealed by a side bias test), irrespective of the location of cue light illumination (Fig. 1b). The shift was repeated three times with the same group of rats. Relative to APO-UNSUS rats, APO-SUS rats made significantly more errors until the criterion (streak of ten correct trials) was reached during the second and third shifts (independent samples *t*-test shift 1 *t* = −0.141, *p* = 0.899, df = 36; shift 2 *t* = 3.409, *p* = 0.002, df = 30.405; and shift 3 *t* = 2.066, *p* = 0.048, df = 30.037), suggesting reduced mPFC-dependent cognitive flexibility in APO-SUS rats. The increased number of errors made by APO-SUS rats represented perseverative errors, indicating a lack of inhibition of previously learned behavior (independent samples *t*-test perseverative errors: *t* = 3.638, *p* = 0.001, df = 36; regressive errors: *t* = 1.362, *p* = 0.182, df = 36; and never-reinforced errors: *t* = 0.096, *p* = 0.924, df = 36).

To investigate whether the observed cognitive impairment is also found with behavioral tests dependent on other frontal areas, such as the orbitofrontal cortex, we tested reversal learning in both the T-maze and the operant cages^34,35^. In the T-maze, rats were trained to retrieve a reward from one arm until they reached a criterion of 70% correct trials per session (Fig. 1c). During the next session, the reward could be retrieved from the opposite arm, as such requiring a complete reversal. The number of trials needed by the APO-SUS and APO-UNSUS rats to reach the criterion of 70% correct performance per session during reversal learning was not significantly different (independent samples *t*-test *t* = 1.993, *p* = 0.068, df = 13). We confirmed this result in the operant setup, where rats were trained to press the lever on one side of the cage until they reached a criterion of a streak of ten correct trials (Fig. 1d). A complete reversal was introduced during the next session, when rats were required to press the lever on the opposite side of the operant cage. This reversal learning was performed three times in a row with the same group of rats. For all three reversals, we found no significant difference in the number of errors made until the APO-SUS and APO-UNSUS rats reached a criterion of a streak of ten correct trials (independent samples *t*-test reversal 1: *t* = 1.579, *p* = 0.123, df = 36; reversal 2: *t* = 1.724, *p* = 0.093, df = 36; and reversal 3: *t* = 0.628, *p* = 0.534, df = 36). However, APO-SUS rats made significantly more perseverative errors when the total number of errors during all three reversals was assessed (independent samples *t*-test perseverative errors: *t* = 4.310, *p* < 0.0001, df = 36 and regressive errors: *t* = 1.897, *p* = 0.067, df = 30.041). This suggests that orbitofrontal cortex-dependent reversal learning behavior is impaired in APO-SUS rats, but not as severely as mPFC-dependent working memory and cognitive inflexibility. Taken together, these data indicate that APO-SUS rats show cognitive inflexibility that involves mPFC dysfunction in SZ-relevant tasks.

### Reduced myelin-related gene expression in the APO-SUS mPFC

Given that the cognitive inflexibility in APO-SUS rats was observed in tasks that are dependent on mPFC functioning^33,36^, we next investigated neurobiological features of this brain region. It was recently hypothesized that in SZ PFC interneuron dysfunction is associated with dysmyelination and that this plays a pivotal role in the pathophysiology of cognitive symptoms of the disorder. Myelination of interneurons occurs during mPFC maturation around P21 in rodents. We have recently shown that in the mPFC of P21 APO-SUS rats, there is a reduced inhibitory input onto pyramidal cells, whereas a normal number and morphology of interneurons were observed^15^. These interneuron abnormalities during adolescence resemble interneuron defects described in SZ patients^6,37^. To examine whether the APO-SUS interneuron defect is indeed linked to impaired myelination, we investigated the molecular and cellular correlates of myelin development in the mPFC of APO-SUS and APO-UNSUS rats.

**Fig. 2:**
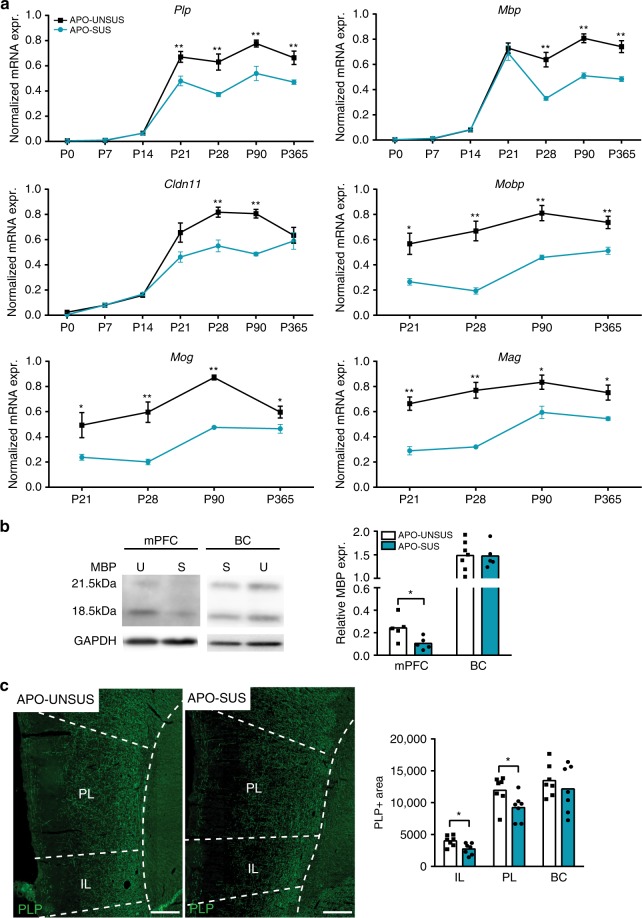
Decreased myelin-related gene expression during the development of APO-SUS versus APO-UNSUS mPFC. **a** Developmental time course of normalized mRNA expression of the myelin-related genes proteolipid protein 1 (*Plp1*), myelin basic protein (*Mbp*), claudin 11 (*Cldn11*), myelin-associated OL basic protein (*Mobp*), myelin OL glycoprotein (*Mog*), and myelin-associated glycoprotein (*Mag*) in mPFC of P0, P7, P14, P21, P28, P90, and P365 APO-SUS rats versus APO-UNSUS rats. *Plp1* P21 **p = 0.006, *Plp1* P28 **p = 0.004, *Plp1* P90 **p = 0.006, *Plp1* P365 **p = 0.008, Mbp P28 **p = 0.001, Mbp P90 **p = < 0.0001, Mbp P365 **p = 0.001, Cldn11 P28 **p = 0.001, Cldn11 P90 **p = < 0.0001, Mobp P21 *p = 0.011, Mobp P28 **p = 0.001, Mobp P90 **p = 0.002, Mobp P365 **p = 0.002, Mog P21 *p = 0.038, Mog P28 **p = 0.001, Mog P90 **p = < 0.0001, Mog P365 *p = 0.040, Mag P21 **p = < 0.0001, Mag P28 **p = < 0.0001, Mag P90 *p = 0.012, and Mag P365 *p = 0.010 in two-tailed independent samples *t*-test with Benjamini–Hochberg multiple comparisons correction**. b** Western blot examples for MBP and GAPDH protein expression in APO-SUS (S) and APO-UNSUS (U) P90 mPFC and BC. Quantification of western blot analysis of MBP normalized to GAPDH in APO-SUS (*n* = 5) and APO-UNSUS rats (mPFC: *n* = 5 BC: *n* = 7) is depicted; *p = 0.033 in two-tailed independent samples *t*-test. **c** Immunohistochemical analysis and quantification of PLP1 protein expression in IL, PL, and BC of P90 APO-SUS (*n* = 7) and APO-UNSUS (*n* = 7) rats. Scale bars 200 µm. IL *p = 0.017 and PL *p = 0.037 in two-tailed independent samples *t*-test. Error bars represent standard error of the mean. Source data are provided as a Source data file.

From P21 onwards, quantitative PCR (qPCR) analysis revealed a decrease in the expression of myelin-related mRNAs encoding proteolipid protein (*Plp)*, myelin basic protein *(Mbp)*, claudin 11 *(Cldn11)*, myelin-associated OL basic protein *(Mobp)*, myelin-associated glycoprotein *(Mag)*, and myelin OL glycoprotein *(Mog)* in the mPFC of APO-SUS versus APO-UNSUS rats (Fig. 2a; for primer design see Supplementary Table 1; for qPCR statistical values and exact number of samples, see Supplementary Table 2). In the barrel cortex (BC) and anterior corpus callosum (CC), we did not observe differences in the mRNA expression levels of any of these genes (Supplementary Fig. 2), indicating unaffected myelin-related gene expression in cortical areas other than the mPFC and in white matter. Additionally, using western blot analysis, we found a decrease in the expression of MBP in adult APO-SUS mPFC, but not BC (Fig. 2b; independent samples *t*-test mPFC: *t* = −2.564, *p* = 0.033, df = 8 and BC: *t* = −0.057, *p* = 0.956, df = 10). PLP immunofluorescence staining also revealed a decrease in myelinated areas in both the infralimbic (IL) and prelimbic (PL) subregions of the mPFC, but not in the BC of adult APO-SUS rats (Fig. 2c, independent samples *t*-test IL: *t* = −2.757, *p* = 0.017, df = 12; PL: *t* = −2.343, *p* = 0.037, df = 12; and BC: *t* = −0.778, *p* = 0.451, df = 12). These results suggest that during the development of the mPFC in APO-SUS rats there is a decrease in myelin-related mRNA expression that persists into adulthood.

### Hypomyelinated parvalbumin interneurons in the APO-SUS mPFC

**Fig. 3:**
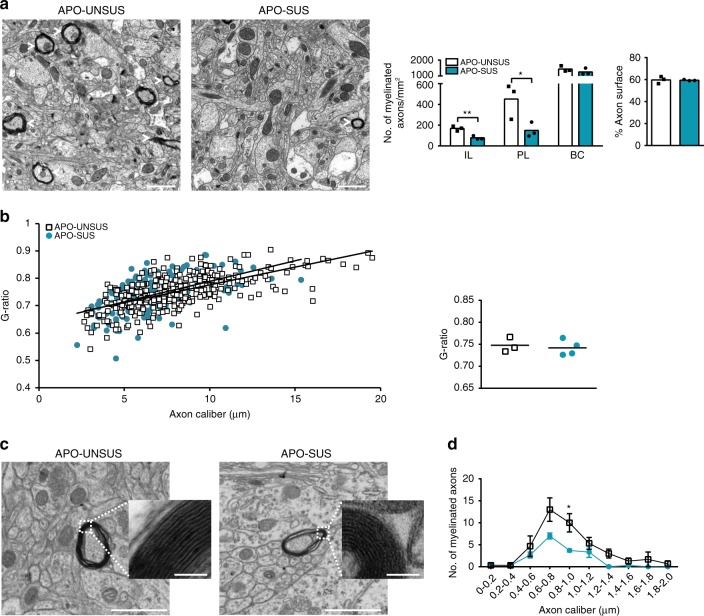
Hypomyelination in APO-SUS versus APO-UNSUS mPFC. **a** Electron microscopy analysis and quantification of the number of myelinated axons in IL, PL, and BC, and the percentage (%) of the total surface that represents axon in PL of APO-SUS (*n* = 3) and APO-UNSUS (*n* = 3) rats. Scale bars represents 1 µm. IL **p = 0.006 and PL *p = 0.047 in two-tailed independent samples *t*-test. **b** G-ratio versus axon caliber and the average G-ratio for all myelinated axons in IL and PL of APO-SUS (*n* = 4), and APO-UNSUS (*n* = 3) rats. **c** Representative high magnification electron microscopy images of the myelin ultrastructure in APO-SUS and APO-UNSUS IL. Scale bars **a**, **c**: 1 µm and 50 nm in insets. Myelin ultrastructure was examined in the IL of *n* = 3 APO-SUS and *n* = 3 APO-UNSUS rats. **d** IL axon caliber frequency distribution in APO-SUS versus APO-USNUS rats. *p = 0.040 in two-tailed independent samples *t*-test. Error bars represent standard error of the mean. Source data are provided as a Source data file.

The observed decreased expression of myelin-related genes in APO-SUS mPFC prompted us to assess the number of myelinated axons and myelin integrity, using transmission electron microscopy at P90. We found a reduced number of myelinated axons in both IL and PL subregions of the mPFC, but not in BC of APO-SUS rats (Fig. 3a; independent samples *t*-test IL: *t* = −5.308, *p* = 0.006, df = 4; PL: *t* = −2.832, *p* = 0.047, df = 4; and BC: *t* = −1.143, *p* = 0.317, df = 4), although axonal density remained unaffected in the APO-SUS versus APO-UNSUS PL (independent samples *t*-test *t* = −0.182, *p* = 0.864, df = 4). This indicates a reduced number of myelinated axons rather than a reduced total number of axons in APO-SUS mPFC. Furthermore, a significant correlation between the thickness of the myelin sheath (G-ratio) and axon caliber was found in mPFC of both APO-SUS and APO-UNSUS rats (Fig. 3b; regression analysis APO-SUS: *F* = 59.830, *p* < 0.001, df = 154 and APO-UNSUS: *F* = 226.528, *p* < 0.001, 327). Note that in APO-SUS mPFC myelin thickness, as measured by its G-ratio, as well as myelin structure was normal (Fig. 3b, c; independent samples *t*-test *t* = −0.431, *p* = 0.684, df = 5), suggesting that no demyelination occurred. The lack of demyelination was further confirmed by the absence of myelin debris in macrophages as revealed by Oil Red O staining in the APO-SUS and APO-UNSUS mPFC (Supplementary Fig. 3). Cumulative distribution analysis of the axon calibers of myelinated axons confirmed a reduction in the number of myelinated axons in APO-SUS IL, and showed that this reduction occurred in axons with a wide range of axon calibers; the 0.8–1.0 μm axon caliber category was significantly different in APO-SUS compared to APO-UNSUS IL, the region where the number of myelinated axons was most significantly decreased in the APO-SUS rat (Fig. 3d; independent samples *t*-test *p* = 0.040, no difference in distribution as revealed by a Chi-square test *p* = 0.482, df = 8). Together, these findings suggest that a number of axons do not get myelinated in the APO-SUS mPFC.

**Fig. 4:**
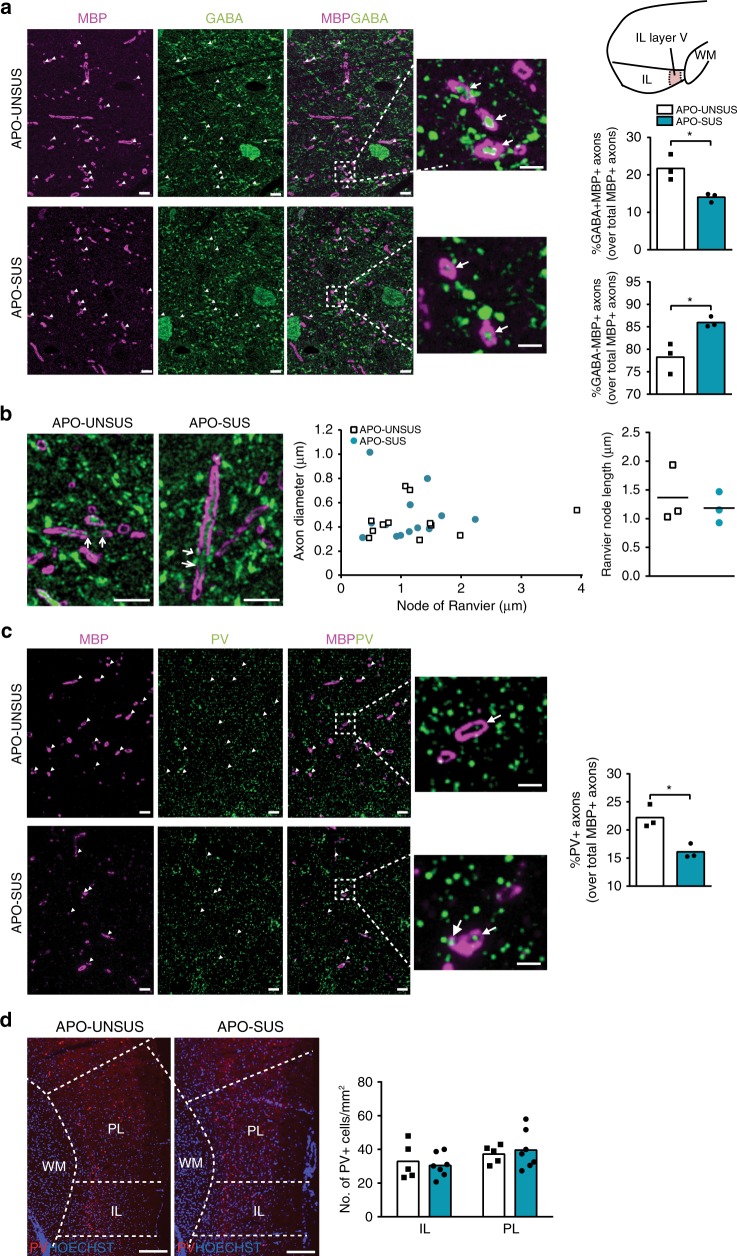
Hypomyelination of GABAergic parvalbumin interneurons in APO-SUS versus APO-UNSUS mPFC. **a** Representative images and quantification of the proportion of myelinated axons (MBP+) that is GABAergic (% GABA+ of total MBP+ axons) or non-GABAergic (% GABA− of total MBP+ axons) in IL layer V of APO-SUS (*n* = 3) versus APO-UNSUS (*n* = 3) rats. Scale bars 5 µm and 2 µm in insets; *p = 0.021 in two-tailed independent samples *t*-test. **b** Representative images and quantification of the length of the nodes of Ranvier of GABAergic myelinated axons in APO-SUS (*n* = 3) and APO-UNSUS rats (*n* = 3). Scale bars 5 µm. **c** Representative images and quantification of the proportion of myelinated axons (MBP+) that are parvalbumin (PV) interneurons (% PV+ of total MBP+ axons). Scale bars 5 µm and 2 µm in insets; *p = 0.013 in two-tailed independent samples *t*-test. **d** Representative images and quantification of the number of PV interneurons/mm^2^ in APO-SUS (*n* = 7) and APO-UNSUS (*n* = 5) mPFC. Scale bars 200 µm. Source data are provided as a Source data file.

We next investigated whether this group of unmyelinated axons in APO-SUS mPFC corresponds to interneurons by performing immunofluorescence co-staining for MBP and GABA on 70 nm ultrathin IL sections. This technique permits visualization of MBP+ myelin sheaths surrounding axons of GABAergic inhibitory neurons, allowing the precise quantification of myelinated inhibitory interneurons at the ultrastructural level (Fig. 4a). We found that the proportion of MBP+ axons that was also GABA+ amounted to 20% in the mPFC of APO-UNSUS rats, similar to previously published values^11^, and this proportion was significantly smaller in APO-SUS mPFC (Fig. 4a; independent samples *t*-test *t* = −3.685, *p* = 0.021, df = 4). Therefore, the percentage of non-GABAergic myelinated axons was higher in APO-SUS mPFC (Fig. 4a; independent samples *t*-test *t* = −3.685, *p* = 0.021, df = 4), and the reduced number of APO-SUS myelinated axons can be primarily attributed to a reduced number of myelinated GABAergic interneurons in the mPFC of APO-SUS versus APO-UNSUS rats. This notion is strengthened by our finding that the number of myelinated axons of different calibers was reduced in APO-SUS IL; myelinated GABAergic interneurons are known to have a range of different axon calibers^11^. To confirm that myelin integrity of GABAergic interneurons was not affected, we measured the length of nodes of Ranvier. We found for the myelinated GABAergic axons a similar average node of Ranvier length of 1.18–1.36 μm in APO-SUS and APO-UNSUS IL (Fig. 4b; independent samples *t*-test *t* = −0.561, *p* = 0.605, df = 4), in line with previously reported values^38^. Myelination of GABAergic interneurons in the PFC has been reported to occur only on parvalbumin interneurons^11,39^. Immunostaining for parvalbumin and MBP on ultrathin tissue sections indeed revealed a reduced number of myelinated parvalbumin axons in APO-SUS relative to APO-UNSUS IL (Fig. 4c; independent samples *t*-test *t* = −4.306, *p* = 0.013, df = 4), confirming parvalbumin interneuron hypomyelination in the APO-SUS mPFC. This was not caused by a decrease in the number of parvalbumin interneurons in the mPFC of APO-SUS rats (Fig. 4d; independent samples *t*-test IL: *t* = −0.467, *p* = 0.650, df = 10 and PL: *t* = 0.436, *p* = 0.672, df = 10).

### OL maturation is impaired in APO-SUS mPFC

**Fig. 5:**
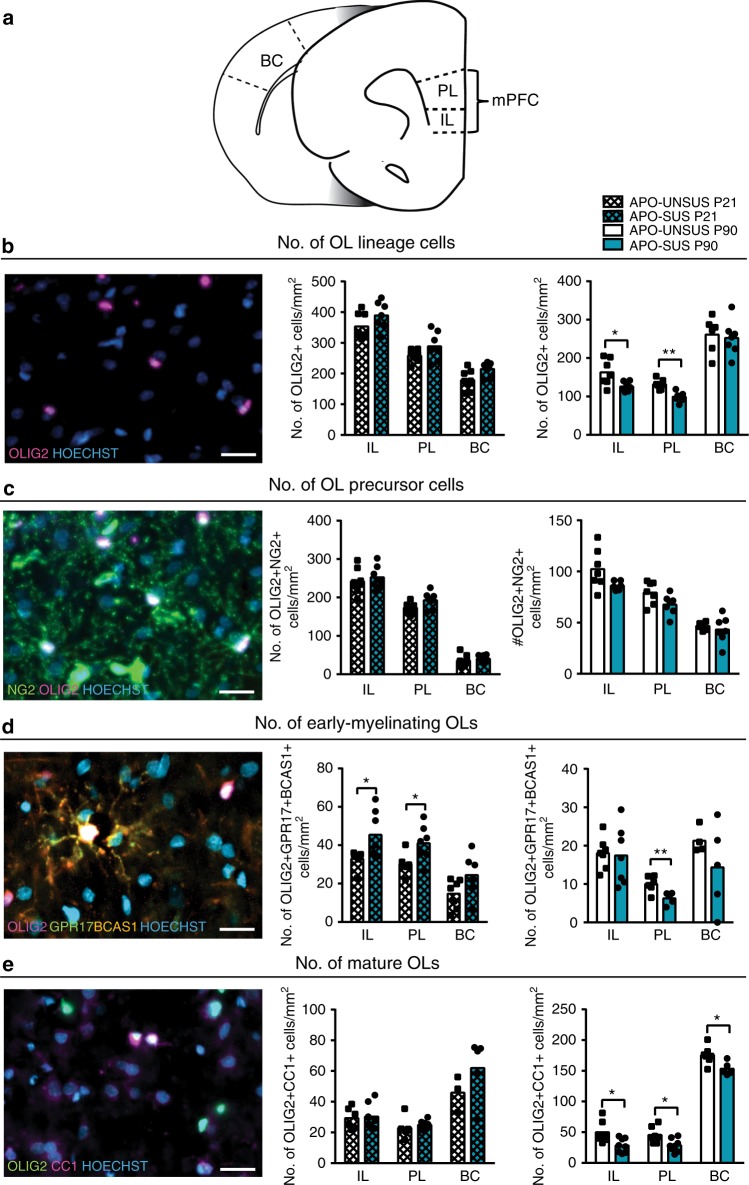
OL maturation impairment in APO-SUS versus APO-UNSUS mPFC. **a** Schematic representation of IL, PL, and BC in rat brain, adapted from the Paxinos and Watson rat brain atlas. **b** Number of OLIG2+ OL lineage cells/mm^2^, P90 IL *p = 0.030 and P90 PL **p < 0.0001 in two-tailed independent samples *t*-test. **c** Number of OLIG2+NG2+ OL precursor cells/mm^2^. **d** Number of OLIG2+GPR17+BCAS1+ early-myelinating OLs/mm^2^, P21 IL *p = 0.038, P21 PL *p = 0.012, and P90 PL **p = 0.010 in two-tailed independent samples *t*-test. **e** Number of OLIG2+CC1+ OLs/mm^2^, P90 IL *p = 0.015, P90 PL *p = 0.015, and P90 BC *p = 0.017. Cell counts in the IL, PL, and BC of APO-UNSUS rats versus APO-SUS rats (P21 *n* = 3–8 and P90 *n* = 5–8 specified in the graphs). Scale bars 20 µm. Source data are provided as a Source data file.

As we identified a lower number of myelinated inhibitory axons, we next examined whether the development of the myelin-producing OLs was impaired in APO-SUS mPFC. Therefore, we assessed the number and maturation state of oligodendroglial cells in APO-SUS and APO-UNSUS mPFC and BC during postnatal development. At P21, when myelination of the mPFC commences, we observed no differences in the number of OLIG2+ OL lineage cells, OLIG2+NG2+ OL precursor cells (OPCs) and OLIG2+CC1+ OLs (Fig. 5a–c, e), whereas we found an increase in the number of OLIG2+GPR17+BCAS1+ early-myelinating OLs (Fig. 5c; independent samples *t*-test IL: *t* = 2.501, *p* = 0.038, df = 7.568 and PL: *t* = 2.898, *p* = 0.012, df = 13), suggesting abnormalities in OL lineage progression already when mPFC myelination starts. In adulthood at P90, we found a decrease in the number of OLIG2+ OL lineage cells in the IL and PL of APO-SUS rats (Fig. 5b; independent samples *t*-test P90 IL: *t* = −2.662, *p* = 0.030, df = 7.502 and PL: *t* = −4.767, *p* < 0.0001, df = 12). Furthermore, a trend toward a decreased number of OLIG2+NG2+ OPCs in the IL and PL of P90 APO-SUS rats was identified (Fig. 5c; independent samples *t*-test P90 IL: *t* = -2.157, *p* = 0.069, df = 6.729; PL: *t* = −2.099, *p* = 0.058, df = 12). Additionally, we observed a significant decrease in the number of OLIG2+GPR17+BCAS1+ early-myelinating OLs (refs. ^40,41^; Fig. 5d; independent samples *t*-test P90 IL: *t* = −0.183, *p* = 0.858, df = 12 and PL: *t* = −3.254, *p* = 0.010, df = 9; see Supplementary Fig. 4 for evidence regarding the specificity of the anti-GPR17 antibody) and a decrease in the number of OLIG2+CC1+ OLs (Fig. 5e; independent samples *t*-test P90 IL: *t* = −2.817, *p* = 0.015, df = 13 and PL: *t* = −2.797, *p* = 0.015, df = 13), indicating that OL maturation is impaired in the adult APO-SUS mPFC. In the APO-SUS BC, we found only a decrease in the number of OLIG2+CC1+ OLs (Fig. 5e; independent samples *t*-test *t* = −2.857, *p* = 0.017, df = 10), suggesting that the OL maturation impairment is more severe in the mPFC than the BC of APO-SUS rats.

### No intrinsic defects of APO-SUS OL lineage progression

**Fig. 6:**
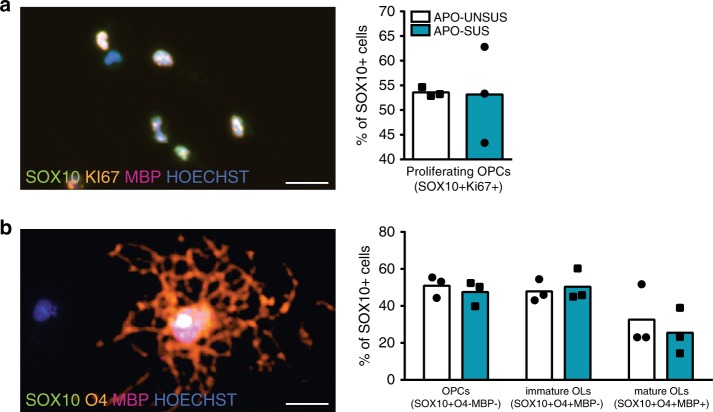
No difference in the intrinsic maturation capability of APO-SUS and APO-UNSUS OPCs. **a** SOX10, Ki67, and MBP co-immunolabeling of APO-SUS and APO-UNSUS primary oligodendroglial cell cultures, and quantification of the percentage of SOX10+ cells that are actively proliferating (SOX10+KI67+), *n* = 3 for each group. **b** SOX10, O4, and MBP co-immunolabeling of APO-SUS and APO-UNSUS primary oligodendroglial cell cultures, and quantification of the percentage of SOX10+ oligodendroglia cells that is OPC (SOX10+O4−MBP−), immature OL (preOL, SOX10+O4+MBP−), and mature OL (SOX10+O4+MBP+), *n* = 3 for each group. Scale bars 20 µm. Source data are provided as a Source data file.

To decipher the cause of the impaired OL maturation that leads to the hypomyelination in the APO-SUS mPFC, we next examined whether OPCs have a cell-autonomous impairment of proliferation, differentiation, and/or maturation. To test this hypothesis, we used primary oligodendroglial cell cultures from cortex of APO-SUS and APO-UNSUS newborn rats, and found no differences in the intrinsic capacity of OPCs to proliferate (Fig. 6a; independent samples *t*-test *t* = −0.074, *p* = 0.944, df = 4) or differentiate (Fig. 6b; independent samples *t*-test OPCs: *t* = −0.670, *p* = 0.540, df = 4; immature OLs: *t* = 0.423, *p* = 0.694, df = 4; and mature OLs: *t* = −0.591, *p* = 0.586, df = 4). These data indicate that the hypomyelination in APO-SUS mPFC is not due to intrinsic defects of OPC proliferation and differentiation. Rather, the results highlight the critical role of the OL microenvironment in the APO-SUS mPFC that could negatively affect OL maturation and myelination. This notion is strengthened by the fact that during development and in adulthood, we did not observe changes in myelin-related gene expression in brain regions other than the mPFC (Supplementary Fig. 2).

### Environmental enrichment improves myelination in APO-SUS mPFC

The development of OLs and the myelination process can be positively influenced by behavioral experiences, such as voluntary exercise, socializing, and environmental enrichment^42–45^. The effects of behavioral experiences on myelination are thought to be caused by cellular changes, such as an increase in neuronal activity, higher levels of neurotransmitters and growth factors, and differential release of metabolites. Importantly, higher rates of sedentary behavior, less physical activity, and decreased sociability in adolescents at high risk to develop SZ correlate with cognitive symptoms in later stages of SZ (refs. ^46–51^). Based on these findings, we applied an environmental enrichment paradigm in order to favorably influence the development of OLs, myelination, and cognitive flexibility in the APO-SUS rat model.

**Fig. 7:**
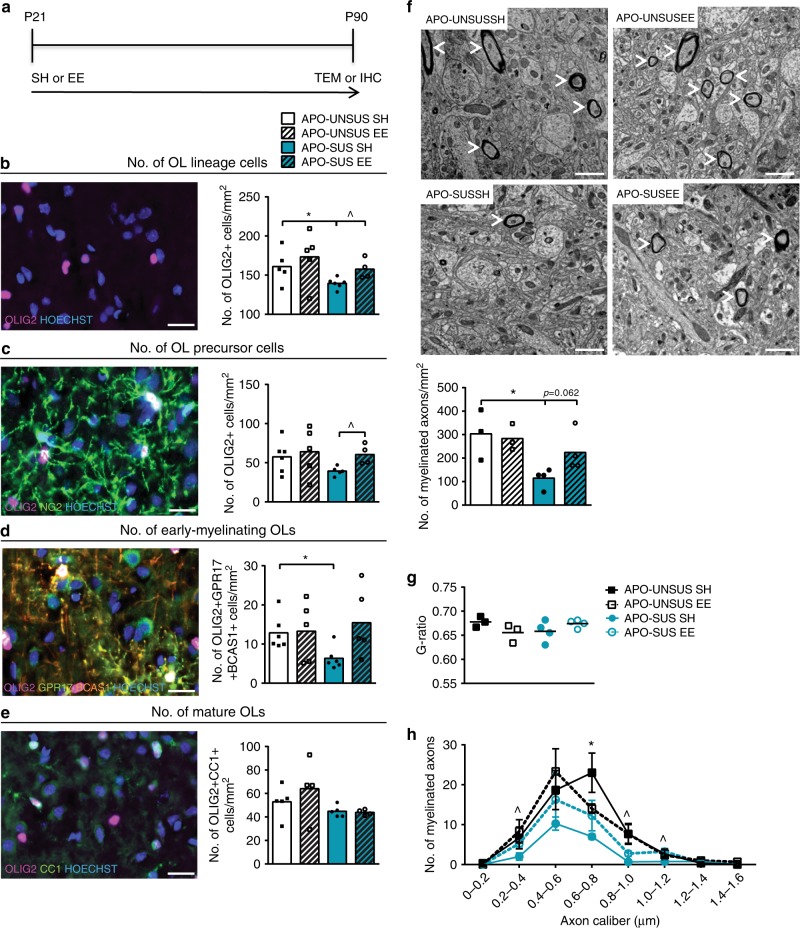
Environmental enrichment improves OL numbers and myelination in the mPFC of APO-SUS rats. **a** Schematic representation of experimental paradigm. APO-SUS and APO-UNSUS rats were housed in standard housing (SH) or in environmental enrichment (EE) conditions starting at P21, and sacrificed at P90 for immunohistochemical (IHC) or transmission electron microscopy (TEM) analyses. **b** Number of OLIG2+ lineage cells; *p = 0.045, ^*p* = 0.018 in two-tailed independent samples *t*-test, **c** number of OLIG2+NG2+ OPCs; ^*p* = 0.013 in two-tailed independent samples *t*-test, **d** number of OLIG2+GPR17+BCAS1+ early-myelinating OLs; *p = 0.012 in two-tailed independent samples *t*-test, and **e** number of OLIG2+CC1+ mature OLs per mm^2^ in the IL subregion of the mPFC of APO-SUS and APO-UNSUS rats in standard housing and environmental enrichment conditions (*n* = 3–6, specified in the graphs). **f** TEM analysis and quantification in the IL of APO-SUS (*n* = 4) and APO-UNSUS (*n* = 3) rats in standard housing and environmental enrichment conditions; *p = 0.021 in two-tailed independent samples *t*-test. **g** Average myelinated axon G-ratio in IL of APO-SUS (*n* = 4) and APO-UNSUS (*n* = 3) rats in standard housing and environmental enrichment conditions. **h** Axon caliber cumulative distributions of myelinated axons in IL of APO-SUS and APO-UNSUS rats in standard housing and environmental enrichment conditions; *p = 0.012; 0.2–0.4 ^*p* = 0.041; 0.8–1.0 ^*p* = 0.047; 1.0–1.2 ^*p* = 0.031 in two-tailed independent samples *t*-test. Scale bars 20 µm in immunofluorescence images and 1 µm in electron micrographs. Error bars represent standard error of the mean. Source data are provided as a Source data file.

We studied OL lineage progression in APO-SUS and APO-UNSUS rats that were reared under environmental enrichment or standard housing conditions from weaning at P21 onward, and were sacrificed at P90 for histological analysis (Fig. 7a). Immunofluorescence analysis revealed that APO-SUS rats reared in an environmental enrichment had significantly more OLIG2+ OL lineage cells in the mPFC than APO-SUS rats reared in standard housing (Fig. 7b; one-way ANOVA *F* = 2.497, *p* = 0.097, df = 3; APO-SUS standard housing versus environmental enrichment *t* = −2.960, *p* = 0.018, df = 8; and APO-SUS standard housing versus APO-UNSUS standard housing *t* = −2.326, *p* = 0.045, df = 9); in the environmental enrichment experiment, we have analyzed IL as there were no substantial differences between IL and PL mPFC subregions in our previous analyses (see Figs. 2–5). Environmental enrichment restored the number of OLIG2+NG2+ OPCs in APO-SUS IL to the IL OPC numbers in APO-UNSUS rats housed under standard conditions (Fig. 7c; one-way ANOVA *F* = 1.472, *p* = 0.260, df = 3; APO-SUS standard housing versus environmental enrichment *t* = −3.055, *p* = 0.038, df = 3.957; and APO-SUS standard housing versus APO-UNSUS standard housing *t* = −1.897, *p* = 0.090, df = 9). There was a trend toward an increase in the number of OLIG2+GPR17+BCAS1+ early-myelinating OLs in the IL of APO-SUS rats reared in environmental enrichment, as compared to APO-SUS rats housed under standard conditions. The number of early-myelinating OLs in IL of APO-SUS rats housed under environmental enrichment conditions was not different from that of APO-UNSUS rats (Fig. 7d; one-way ANOVA *F* = 2.321, *p* = 0.110, df = 3; APO-SUS standard housing versus environmental enrichment *t* = −2.291, *p* = 0.80, df = 4.275; and APO-SUS standard housing versus APO-UNSUS standard housing *t* = −3.049, *p* = 0.012, df = 10). The number of OLIG2+CC1+ OLs, however, was not affected by environmental enrichment in the mPFC of both APO-SUS and APO-UNSUS rats (Fig. 7e; one-way ANOVA: *F* = 1.770, *p* = 0.199, df = 3; APO-SUS standard housing versus environmental enrichment *t* = 0.326, *p* = 0.754, df =7 ; and APO-SUS standard housing versus APO-UNSUS standard housing *t* = −1.270, *p* = 0.240, df = 8). These data indicate that environmental enrichment led to a significantly increased number of OL lineage cells and OPCs, and a trend toward an increased number of early-myelinating OLs, while the number of mature OLs remained unaffected in APO-SUS mPFC.

We next investigated whether an environmental enrichment-induced rescue of the numbers of OL lineage cells, OPCs and early-myelinating OLs would be sufficient to restore myelination of interneurons in APO-SUS mPFC. Ultrastructural analysis revealed that in the APO-SUS mPFC environmental enrichment restored the number of myelinated axons to APO-UNSUS standard housing level, while environmental enrichment had no effect on the number of myelinated axons in APO-UNSUS mPFC (Fig. 7f; one-way ANOVA *F* = 4.551, *p* = 0.029, df = 3; APO-SUS standard housing versus environmental enrichment *t* = −2.291, *p* = 0.062, df = 6; and APO-SUS standard housing versus APO-UNSUS standard housing *t* = −3.302, *p* = 0.021, df = 5). The G-ratio of APO-SUS and APO-UNSUS myelinated axons was not affected by environmental enrichment (Fig. 7g; one-way ANOVA *F* = 1.622, *p* = 0.246, df = 3), and chi-square test on cumulative axon caliber distribution showed that there were no differences in the distribution of myelinated axon caliber between the groups (Fig. 7h; chi-square test APO-SUS standard housing versus environmental enrichment *p* = 0.338 and APO-SUS standard housing versus APO-UNSUS standard housing *p* = 0.214). However, environmental enrichment significantly increased the number of myelinated axons of 0.2–0.4 μm, 0.8–1.0 μm, and 1.0–1.2 μm axon caliber in APO-SUS mPFC to control levels (Fig. 7h; independent samples *t*-test *t* = −2.600, *p* = 0.041, df = 6; *t* = −2.926, *p* = 0.047, df = 5; and *t* = −2.810, *p* = 0.031, df = 6, respectively in APO-SUS standard housing versus environmental enrichment). We conclude that environmental enrichment during mPFC development restored the number of OL lineage cells, OPCs, and early-myelinating OLs, and improved the myelination of interneurons in the APO-SUS mPFC.

### Environmental enrichment improves cognition of APO-SUS rats

**Fig. 8:**
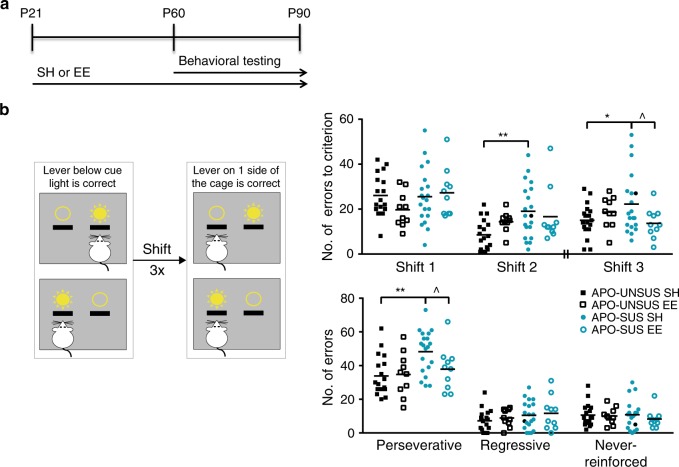
Environmental enrichment improves cognitive inflexibility of APO-SUS rats. **a** Schematic representation of experimental paradigm. APO-SUS and APO-UNSUS rats were housed in standard housing or in environmental enrichment conditions starting at P21, and subjected to behavioral testing between P60 and P90. **b** Schematic representation of the extra-dimensional set-shifting task. Rats were trained to press the lever above which a cue light was illuminated. From trail 21 of the next session onward, rats were required to press the lever on one side of the cage irrespective of the cue light. Number of errors to criterion (streak of ten correct trials) and total number of perseverative, regressive, and never-reinforced errors in APO-UNSUS (*n* = 18) and APO-SUS (*n* = 20) rats in standard housing (the same data as are presented in Fig. 1b) and environmental enrichment (*n* = 10) are depicted. Note that between shift 2 and shift 3 the reversal learning trials were conducted (see Supplementary Fig. 5). Shift 2 **p = 0.001, shift 3 *p = 0.028, shift 3 ^*p* = 0.030, perseverative errors **p = 0.001, ^*p* = 0.036 in two-tailed multivariate ANOVA post hoc testing. Source data are provided as a Source data file.

Finally, we assessed whether the rescue of interneuron myelination in the mPFC of APO-SUS rats would be sufficient to restore cognitive inflexibility in the extra-dimensional set-shifting test. To answer this question, APO-SUS and APO-UNSUS rats were reared in environmental enrichment or standard housing from weaning at P21 onward, and behavioral testing was performed between P60 and P90 (Fig. 8a, Supplementary Fig. 5). Multivariate ANOVA revealed a significant effect of the behavioral treatment on the number of errors until criterion (streak of ten correct trials) during extra-dimensional set-shifting, as well as on the number of perseverative errors (Fig. 8b; *F* = 2.529, *p* = 0.011, df =9 and *F* = 2.367, *p* = 0.017, df =9). Post hoc testing confirmed a significant improvement in the number of errors until criterion in the third extra-dimensional shift and a reduction of the total number of perseverative errors in APO-SUS rats reared in an environmental enrichment. APO-UNSUS rats reared in an environmental enrichment compared to standard housing-reared APO-UNSUS rats showed a similar number of perseverative errors during extra-dimensional set-shifting, but a decreased error number in the first and an increased error number in the second shift. The number of perseverative errors made during reversal learning was also significantly reduced in APO-SUS rats (Supplementary Fig. 5). Thus, environmental enrichment reversed behavioral inflexibility in APO-SUS rats.

## Discussion

In this study, we have provided direct evidence that in a rat model with SZ-relevant features interneurons are hypomyelinated in the mPFC, and that restoring interneuron myelination through environmental enrichment applied during mPFC development improves cognitive flexibility. We found mPFC-dependent cognitive inflexibility in both spatial working memory and extra-dimensional set-shifting. Spatial working memory is also impaired in SZ patients^52^ and has been linked to reduced cortical GABAergic signaling^53,54^. APO-SUS rats display spatial working memory impairment on the more difficult trials, indicating a working memory deficit as well as cognitive inflexibility. The extra-dimensional set-shifting task we performed, is a rodent version of the Wisconsin card sorting test that has been used to reveal impaired set-shifting in SZ patients^55^. Interestingly, like in SZ patients the set-shifting impairment in the APO-SUS rats was characterized by a disinhibition of previously learned behavior^56^. Subtle alterations in SZ PFC inhibitory circuits are thought to underlie these set-shifting impairments^37^. The fact that APO-SUS rats showed cognitive inflexibility only when task demand increased, but not on the first extra-dimensional shift, suggests just subtle alterations in mPFC circuit functioning rather than a complete failure to activate the mPFC. Reversal learning, relying more on orbitofrontal cortex and less on mPFC functioning^34^, was also impaired in APO-SUS rats, albeit detectable only after multiple reversals were performed consecutively. Similarly, in SZ patients perseverative deficits in (probabilistic) reversal learning are found only in a subset of patients and less consistently than abnormalities in extra-dimensional set-shifting^57,58^. Thus, APO-SUS rats show cognitive inflexibility with characteristics reminiscent of cognitive impairment in SZ patients, including features suggestive of alterations in mPFC inhibitory circuits.

Dysfunctioning of interneurons and defective myelination are both well-replicated neurobiological abnormalities observed in SZ, including decreased myelin-related gene expression in postmortem SZ frontal cortex^17,18^. Our data shows that decreased expression of myelin-related mRNAs and proteins starts during adolescence, and persists into adulthood in the APO-SUS mPFC, supporting the hypothesis that in SZ defective myelination occurs during adolescent mPFC development, rather than in adulthood by demyelinating insults. Strengthening this notion is the fact that active demyelination does not occur in the mPFC of APO-SUS rats, and that myelinated fibers in the APO-SUS mPFC have a typical G-ratio and myelin structure, indicating hypomyelination rather than demyelination in the mPFC. Furthermore, ultrastructural analysis revealed a reduced number of myelinated axons in the adult APO-SUS mPFC. Importantly, we found that the population of unmyelinated axons corresponded to parvalbumin interneurons, while the number of parvalbumin interneurons remained the same, demonstrating the occurrence of parvalbumin interneuron hypomyelination in the mPFC of the APO-SUS model with SZ-relevant features. In SZ, parvalbumin interneurons have an intrinsic defect that leads to an impaired inhibitory control and disturbed cognitive functioning^37^. Interestingly, it has been recently suggested that a myelination deficiency of parvalbumin interneurons may underlie defective inhibitory regulation in the PFC (ref. ^14^). We here show that in the mPFC of a rat model of SZ parvalbumin interneurons are indeed hypomyelinated.

A plausible reason for the observed parvalbumin interneuron hypomyelination may be the impaired maturation of OL lineage cells in the mPFC of APO-SUS rats. The increase in the number of early-myelinating OLs at P21 indicates that in APO-SUS mPFC there are abnormalities in OL lineage progression already at the onset of myelination. This was accompanied by decreased myelin-related mRNA expression, suggesting that in the APO-SUS mPFC the OLs produce less myelin-related mRNAs per cell. The fact that at P90 (in adulthood), we found a similar number of OPCs and decreased numbers of early-myelinating, as well as mature OLs points to an impairment of OL maturation, consistent with the significantly reduced expression of all six myelin-related genes examined in the adult APO-SUS mPFC. Likewise, in postmortem PFC of SZ patients, the total number of OL lineage cells is reduced, while no changes in the total number of OPCs have been observed^59,60^. The reduced number of mature OLs in APO-SUS BC was not accompanied by changes in OL differentiation or myelination, and suggests an increased myelin production by mature OLs in this brain region. In contrast, the differences in OL maturation observed in the mPFC do lead to affected myelination. Primary oligodendroglial cell cultures from APO-SUS and APO-UNSUS cortex revealed that the intrinsic capability of APO-SUS OPCs to proliferate and differentiate was still intact. This suggests that APO-SUS oligodendroglia do have the capacity to terminally differentiate and myelinate axons. We therefore conclude that the OL microenvironment within the APO-SUS mPFC plays a pivotal role in inducing the OL lineage progression impairment observed in the APO-SUS rats.

Behavioral experiences are known to influence the development of oligodendroglia and myelination in the PFC (ref. ^42^). For example, in rodents voluntary exercise increases OL proliferation in the PFC^61^, sociability influences myelination and OL development in the PFC during adolescence^43^, and environmental enrichment stimulates OLs to produce more myelin^44,45^. The effects of behavioral experiences are strongest within the critical period of PFC myelination, i.e., during adolescence^43^. Importantly, adolescent individuals at high risk for SZ show higher rates of sedentary behavior, less physical activity, and decreased sociability that correlate with cognitive symptoms in later stages of SZ (refs. ^46,47,62^). Furthermore, levels of academic achievement and social interactions prior to disease onset during adolescence are associated with the degree of impairment in working memory and executive functioning during later phases of SZ (refs. ^49–51^). This indicates that socializing and cognitive challenges can positively influence cognition during the course of SZ. Indeed, exposing APO-SUS rats to an environmental enrichment paradigm that included voluntary exercise, increased sociability, and opportunity for novelty exploration during adolescent mPFC development restored the number of OL lineage cells. The increased density of OPCs following environmental enrichment suggests that under this condition more OL lineage cells remain in the OPC pool. Other studies that have applied environmental enrichment to treat glial abnormalities in rat brain have reported similar increases in the number of OPCs (refs. ^63–65^). Environmental enrichment did not affect in APO-SUS mPFC the total number of mature OLs, but increased the number of early-myelinating OLs as well as the number of myelinated axons to numbers comparable to those in APO-UNSUS mPFC. This is in line with previous studies in rodents, showing that environmental enrichment increases myelin production^42,44,45^. The exact mechanism underlying the myelin increase is at present unclear, but it has been hypothesized that environmental enrichment causes changes in neuronal activity, axon-OPC synapses, growth factor release, neurotransmitter release, excretion of metabolites, and inflammatory factors that can influence oligodendroglial cells and myelination^42^. Environmental enrichment during mPFC development was also sufficient to rescue cognitive flexibility in the extra-dimensional set-shifting test, clearly indicating that developmental interneuron hypomyelination contributes to the cognitive impairment observed in the APO-SUS rat model of SZ.

In individuals at high risk to develop SZ, environmental enrichment, exercise, and sociability training have the potential to rescue aberrant PFC myelin development. High-risk individuals show a significant decrease in white matter integrity in frontal circuits after transitioning into psychosis^66^, indicating a developmental aspect to psychosis transitions that indeed involves white matter and myelination^22^. Furthermore, behavioral therapy enhances white matter integrity and improves cognition in SZ patients^67,68^. Taken together, the findings suggest that stimulating myelination in the PFC by applying environmental enrichment or other targeted behavioral therapies during adolescence could be an attractive preventive measure for individuals with a high risk of transitioning to SZ.

## Methods

### Animal model

APO-SUS and APO-UNSUS rat lines have been selectively bred from an outbred Nijmegen Wistar rat population. Wistar rats that displayed stereotyped gnawing behavior (>500 gnaws in 45 min) upon injection of apomorphine were selectively bred as APO-SUS rats. The same selective breeding was performed with the rats that showed a weak apomorphine-induced stereotypy (<10 gnaws in 45 min; APO-UNSUS rats). Apomorphine injection and behavioral selection were only performed with the first 15 generations of APO-SUS and APO-UNSUS rats. In the subsequent breedings, APO-SUS rats displayed SZ-relevant features without this pharmacological treatment. In this study, naive male APO-SUS and APO-UNSUS rats from the 38th–43rd generation were used. Rats were housed in pairs in a temperature- and humidity-controlled room with a 12-h light–dark cycle (lights on at 7.00 a.m.), and ad libitum access to water and standard laboratory chow (V1534-703, SSNIFF, Germany), unless otherwise indicated. Animal experiments were approved by the Animal Ethics Committee of Radboud University Nijmegen Medical Centre, Nijmegen, the Netherlands, and were conducted in accordance with Dutch legislation (Herziene Wet op Dierproeven, Art 10.a.2, 2014).

### T-maze behavioral tests

During the continuous delayed alternation test, APO-SUS and APO-UNSUS rats were kept on a food restriction schedule. A limited amount of standard laboratory chow was given to maintain the rats at 85–90% of their free-feeding weight. Rats were habituated for 20 min to a T-maze with food pellets scattered around the maze (arm length 50 cm, arm height 40 cm, and arm width 14 cm with a start box of 15 × 14 × 40 cm that could be closed off by a guillotine door). Food pots (4 × 4 ×4 cm) were present 4 cm from the end of each arm. The animals were trained to perform a minimum of seven trials within 20 min (i.e., retrieving a food pellet from both food pots), after which the continuous delayed alternation paradigm started. Rats were tested during the light phase and performed nine trials per day (i.e., a session) for 10 days. During the start trial of each session, a food pellet was present both in the left and the right arm of the T-maze. The rat was placed in the start box and upon lifting of the guillotine door the rat was allowed to move freely around the maze until one of the food pellets was retrieved. After that the rat was placed back in the start box for the delay period of either 10 or 60 s. In the first test trial only the arm that the animal did not visit during the start trial was baited. During following trials, a food pellet was present only in the arm that was not baited during the previous trial. Hence, rats had to alternate between the left and right arms of the T-maze (Fig. 1a). Trials were considered correct when the first arm entry (defined as having four paws in one arm) was into the baited arm and the start trial of each session was not taken into account during data analysis. For both the 10- and 60-s delay conditions, five sessions were conducted and the average percentages (%) of correct trials for these five sessions were calculated. For T-maze reversal learning, the rats were trained until they could retrieve a food pellet from one arm of the maze with a performance of 70% correct trails per session. During the following session, bait could be retrieved from the opposite arm, hence requiring a complete reversal (Fig. 1c). We counted the number of trials until a performance of 70% correct trials per session was achieved.

### Operant attentional set-shifting tests

Upon the start of behavioral training, rats were food restricted and received 5–8 g of food per 100 g of rat daily. Rats were pre-exposed once to grain reward pellets (Rodent Tablet [5TUM], 45 mg, TestDiet, USA) in the home cage. Operant conditioning chambers (29.5 cm: L, 24 cm: W, 25 cm: H; Med Associates, Georgia, VT) were situated in light and sound-attenuating cubicles equipped with a ventilation fan. Each chamber was equipped with two 4.8-cm-wide retractable levers, placed 11.7 cm apart and 6 cm from the grid floor. A cue light (28 V, 100 mA) was present above each lever. At the same wall, a reward pellet could be delivered in a magazine between the levers and a house light (28 V,100 mA) was located on the same wall. The lever presentation and cue light illumination sides were counterbalanced between rats. Operant extra-dimensional set-shifting and reversal learning procedures were programmed, and analyzed in MED-PC V. Procedures are based on previous reports^33^ and described below.

During pretraining, the rats were first habituated to the operant cage and received 50 food pellets with an inter-trial interval varying between 10 and 50 s. During the second training phase, one of the levers was extended during the entire session and each lever-press resulted in the delivery of a reward pellet. After the criterion of 50 lever presses was reached, this was repeated with the other lever. During the third training phase, rats were familiarized with the house light and the insertion of the levers into the chamber, and were required to press within 10 s to receive a food pellet. Upon pressing, the lever retracted, a reward pellet was delivered, and the house light remained illuminated for another 4 s. If the rat did not press the lever within 10 s, the lever retracted and the house light went off. In each pair of trials, the left or right lever was presented once, and the order within the pair of trials was random. Five of these retractable lever-press training sessions were performed, each consisting of 90 trials with 20 s inter-trial intervals. Immediately after the last session of retractable lever-press training, without being removed from the operant chamber, the rats performed a side bias test. During the side bias test both levers were presented and rats received a food pellet upon pressing either the left or the right lever. Upon pressing, the levers retracted, a reward pellet was delivered, and the house light remained illuminated for another 4 s. In case the rat did not press a lever within 10 s, the levers retracted and the house light went off. Subsequently, a food pellet was delivered only upon pressing the lever that was not chosen during the previous trial. In case the rat pressed the same lever as during the previous trials no food pellet was delivered, the levers retracted and the house light turned off. Trials continued until the rat pressed the lever that was not chosen during the initial trial. After pressing both levers, a new set of trials commenced and seven sets of trials were applied. The number of responses on each lever was used to determine side preference.

Upon completion of pretraining, the rats were trained to perform visual cue discrimination. The cue light was randomly presented above the left or right lever for 3 s, then both levers extended and the house light turned on. The rat was required to press the lever above which the cue light was illuminated within 10 s in order to receive a reward pellet. When rats reached the criterion of ten subsequent correct trials, the session finished. During consecutive phases of the experiment, rats were either required to perform an extra-dimensional set-shift or a reversal learning procedure. During extra-dimensional set-shifting, the first 20 trials of the session consisted of the ‘old rule’, in which rats had to press the lever above which a cue light was illuminated, and from trial 21 onward, rats were required to press their non-preferred lever (as indicated by the side bias test) irrespective of the cue light illumination (Fig. 1b). During reversal learning, the first 20 trials consisted of pressing the rats’ non-preferred lever, and from trial 21 onwards, rats were required to press the opposite lever to receive a reward pellet (Fig. 1d). Rats performed two extra-dimensional set-shifts, then three reversal learning shifts, and then another extra-dimensional set-shift. We measured the number of errors made until criterion of ten subsequent correct trials was reached, and classified the errors as perseverative errors (following the ‘old rule’), regressive errors (following the ‘old rule’ while >70% of previous trials were correct), or never-reinforced errors (pressing a lever that was incorrect during both the ‘old rule’ and during the current rule).

### Environmental enrichment

Rats were weaned at P21 and placed in environmental enrichment or standard housing conditions. For environmental enrichment, rats were housed in groups of ten animals in 100 × 54.5 × 48 cm cages with cage enrichment in the form of toys, running wheels, tunnels, and nesting places. Enrichment was of different colors and textures, and was changed three times a week to promote exploration behavior and rats were handled once a week. Rats in standard housing were housed in pairs in standard cages with standard enrichment in the form of a rat retreat. Rats in standard housing and environmental enrichment conditions were housed in the same room. Rats were either sacrificed at P90 for immunohistochemistry or transmission electron microscopy experiments, or did the operant attentional set-shifting task starting at P60.

### Micropunching and RNA isolation

Naive P0, P7, P14, P21 (±1 day), P60 (±1 day), P90 (±1 day), P120 (±1 day), and P365 (±14 days) APO-SUS and APO-UNSUS rats were sacrificed by direct decapitation, and brains were isolated, frozen on dry ice, and stored at −80 °C. Brains of P0, P7, and P14 were freshly dissected, while brains of P21, P60, P90, P120, and P365 were micropunched. Micropunching was performed in a cryostat (Leica) at −15 °C and the Paxinos and Watson rat brain atlas was used to aid dissection. mPFC was collected with a 1.20 or 2.00-mm punch needle (Harris) from 300 µm coronal sections at Bregma 4.00–2.20. CC was microdissected at room temperature (RT) and striatum (STR) punched with a 2.00-mm punch needle from Bregma 1.60 to −0.20. BC was punched with a 2.00-mm needle at Bregma 8.08–6.10. Dissected tissues were stored at −80 °C until further analysis. Tissue samples were homogenized using Trizol (Sigma) and RNA was extracted with chloroform, precipitated with isopropanol and glycogen (Fermentas), washed with 75% ethanol, dissolved in MilliQ H2O, and stored at −80 °C until further analysis.

### Quantitative PCR

For qPCR analysis, RNA samples were treated with DNase I (Fermentas) and cDNA was synthesized using the Revert Aid H-minus first strand cDNA synthesis kit (Thermo Scientific). cDNA was diluted in MilliQ H2O and stored at −20 °C. qPCR samples were pipetted using a robot (Corbett Robotics) and contained 2.0 μL diluted cDNA, 0.8 μL 5 μM forward primer, 0.8 μL 5 μM reverse primer, 5 μL SybrGreen mix (Roche), and 1.8 μL MilliQ H2O. qPCR was performed with a Rotor Gene 6000 Series (Corbett Life Sciences) using a three-step paradigm with a fixed gain of eight. Forty five to 50 cycling steps of 95, 60, and 72 °C were applied and fluorescence was acquired after each cycling step. Primers were designed with NCBI Primer-Blast or Primer Express 2.0 and synthesized by Sigma (for primer pair sequences, see Supplementary Table 1). Take off and amplification values of the housekeeping genes (Ywhaz, β-actin, Ppia, and Gapdh) were used to determine the normalization factor with GeNorm 39 (ref. ^69^) after which normalized mRNA expression levels were calculated.

### Western blot analysis

For western blot analysis, tissue samples were homogenized in RIPA buffer (150 mM NaCl, 1% NP40, 0.1% SDS, 0.5% Na-deoxycholate, 50 mM Tris-HCL pH 8.0, and protease inhibitor) using 20 (mPFC and BC) or 25 (CC) strokes in a glass potter, and protein levels were determined with a BCA assay (Thermo Scientific). A total of 80 µg of mPFC, 5 µg of CC, and 30 µg of BC protein were used for SDS–PAGE and western blotting onto PVDF membranes. Blots were blocked in 1× PBS, 0.1% Tween, and 10% non-fat milk (Elk, Campina) and incubated with MBP antibody (MAB386, Millipore) at 1:4000 or 1:1000, and GAPDH antibody (2118, Cell Signaling) at 1:5000 in 1× PBS, 0.1% Tween, and 5% milk at 4 °C overnight. Secondary antibodies (anti-mouse or anti-rabbit peroxidase; GAM/IgG(H + L)/PO and GAR/IgG(H + L)/PO, Nordic Immunology) were incubated for 2 h at RT at 1:5000, and images were acquired using Lumi-Light Western blotting substrate (Sigma) in an ImageQuant LAS-4000 digital imaging system (GE Healthcare) and analyzed using FIJI.

### Immunohistochemistry

For immunohistochemistry, naive APO-SUS and APO-UNSUS rats of P21 (±1 day) and P90 (±1 day) were perfused with 2% paraformaldehyde (PFA). Perfused brains were removed, postfixed overnight, and placed in 30% sucrose in PBS for 3–5 days, frozen on dry ice and stored at −80 °C until further processing. Coronal cryosections of 10–14 μm were collected in a cryostat (Leica) and rehydrated in 1× PBS, 0.1% Triton X-100, or for OLIG2 NG2 immunohistochemistry in 1× PBS, 0.05% Tween-20. For OLIG2-CC1 staining antigen retrieval was performed in a microwave using citric acid-based antigen unmasking solution (Vector). Tissue was blocked in 4% BSA, 0.1% Triton X-100 or 5% NGS/NDS/NHS, 1% BSA, 1% glycine, 0.1% lysine, and 0.4% triton X-100 for 1 h at RT. Primary antibodies were anti-OLIG2 (AB9610, Millipore 1:1000, ab109186, Abcam 1:400, or MABN50, Millipore 1:1000), anti-CC1 (OP80, Calbiochem 1:100), anti-NG2 (MAB5384, Millipore 1:200 or AB5320, Millipore 1:100), anti-GPR17 (10136, Cayman Chemical 1:100), anti-BCAS1 (sc-136342, Santa Cruz 1:100), or anti-PLP (MCA839G, Biorad 1:200) and incubated overnight at 4 °C. Secondary antibodies were 488, 555, 568, and 647-conjugated anti-rabbit or anti-mouse (IgG, IgG2a, and IgG2b, Alexa 1:1000) or TRITC-conjugated anti-mouse (IGg1, Southern Biotech 1:100), and incubated for 1–2 h at RT. Hoechst (H6024, Sigma 1:1000) was added as a nuclear counterstain in MilliQ H2O, rinsed with 60% isopropanol, and incubated in 60% isopropanol with 0.3% Oil red O (O0625, Sigma) for 15 min at RT, and rinsed with 60% isopropanol and MilliQ H2O. As positive control for Oil red O staining, a mouse spinal cord lesion with active demyelination induced by experimental autoimmune encephalomyelitis was used. Sections were mounted in Fluoromount (0100-01, Southern Biotech) and 20× images were obtained using an Axioscan (Zeiss) and analyzed with FIJI. Regions of interest were drawn and all cells in the region of interest were counted manually in one to three brain sections for OLIG2+NG2+, OLIG2+GPR17+BCAS1+, and OLIG2+CC1+ cells. OLIG2+ cell counts were obtained in the OLIG2−NG2 or OLIG2−CC1 images. Positive areas for PLP and Oil red O were quantified using a threshold of 25/255 and 0/133, respectively. For PLP images, background was subtracted with a rolling ball radius of 50 pixels.

### Electron microscopy

For electron microscopy, naive animals of P90 (±1 day) were perfused with 2% PFA/2% glutaraldehyde and perfused brains were removed, postfixed overnight in 2% PFA/2% glutaraldehyde, and stored at 4 °C in PBS/0.01% azide until further processing. Sagittal sections of 100 μm were collected using a vibratome (Leica), fixed with 2% osmium tetroxide, and contrast was obtained with 5% uranyl acetate. Following ethanol dehydration, sections were embedded in epon resin and ultrathin (70–100 nm) sections were obtained with an ultramicrotome (Leica). Sections were contrasted using lead citrate and 40 non-overlapping 26,000× images were obtained in each region of interest. Myelinated axons were counted in all 40 images per brain region, and the G-ratio and axon caliber of all myelinated axons perpendicular to the field of view were measured in FIJI. The percentage of axon surface was calculated using 49 equally (200 µm) spaced crosses superimposed over ten randomly picked images. The percentage of crosses that touched an axon was calculated.

### Immunofluorescence staining of ultrathin sections

Naïve APO-SUS and APO-UNSUS rats of P90 (±1 day) were perfused with 2% PFA/2% glutaraldehyde and perfused brains were removed, postfixed overnight in 2% PFA/2% glutaraldehyde, and stored at 4 °C in PBS/0.01% azide until further processing. Sagittal sections of 100 μm were collected using a vibratome (Leica), and IL was dissected according to Figure 81 of the Paxinos and Watson Rat Brain Atlas. IL samples were dehydrated in ethanol (50%, 70%, 80%, and twice 90% ethanol for 15 min), embedded in LRwhite resin (Electron Microscopy Sciences) at RT for 14 h, and polymerized in LRwhite resin in airtight capsules at 60 °C for 2–3 days. Ultrathin (70–100 nm) sections were obtained with an ultramicrotome (Leica), collected on gelatin-coated glass slides, and stained with anti-MBP (MBP, Aves 1:200) and anti-GABA (AB175, Millipore 1:500), or anti-parvalbumin (PV27 Swant 1:300) antibodies using a protocol based on refs. ^11,38^ with adjustments. Sections were immersed in 100 mM glycine in milliQ water, washed with 1× PBS, and primary antibodies diluted in 2% BSA in 1× PBS were applied at 4 °C overnight. Sections were then washed with 1× PBS, secondary antibodies (Alexa anti-chicken 594, anti-Rabbit 488, and anti-guinea pig 488 1:150 in 1% BSA and in 1× PBS) were added for 30 min at RT, and sections were subsequently washed with 1× PBS and coverslipped using Fluoromount (Thermo Fisher). Images were obtained from IL layer V, 300 µm from the CC, using a wide-field fluorescent microscope with a 63× oil lens (Leica) and Zen 2 (Leica, blue edition) software. The number of MBP+GABA+ or MBP+parvalbumin+ axons, as well as the total number of MBP+ axons was quantified. Because of the reduction in the number myelinated axons in APO-SUS mPFC, we calculated the percentage of MBP+ axons that was also GABA+ or PV+. Nodes of Ranvier were measured in Zen software in MBP+GABA+ axons that were horizontal to the field of view.

### Primary oligodendroglial cell cultures

Mixed glia cultures were obtained from cortices of P1 APO-SUS and APO-UNSUS rats, *n* = 3 replicates per condition. The tissue was homogenized in mixed glia culture medium (GlutaMAX (Invitrogen), 10% fetal bovine serum (Thermo Fisher), 1% Pen-Strep (Thermo Fisher), and 1% non-essential amino acids (Thermo Fisher)) and cells were kept in this medium on 1:10 poly-L ornithine-coated T75 flasks at 37 °C 5% CO2. Medium was refreshed after 7 and 13 days in culture, and at day 14 OPCs were purified using a shaking protocol. Mixed glia cultures were shaken at 7 × *g* for 1 h to remove microglia cells, then cultures were shaken again at 7 × *g* for 18 h to purify OPCs. Supernatant containing OPCs was placed on petri dishes (Falcon) for 3 × 5 min to eliminate astrocyte contamination. OPCs were then plated on PLO-coated coverslips in 24 well plates. After 4 h, medium was changed to either proliferation medium (DMEM-F12 (Invitrogen), 0.5% B27 (Sigma) 1% Pen-Strep (Thermo Fisher), 0.05% fibroblast growth factor (25 ng/ml; Sigma), and 0.05% platelet derived growth factor BB (10 ng/ml; Sigma)), or differentiation medium (DMEM-F12 (Invitrogen), 0.5% B27 (Sigma), 1% penicillin–streptomycin (Thermo Fisher), and 0.05% of T3 (thyroid hormone, 40 ng/ml, Sigma)) for 4 days. After 4 days, cultures were stained with O4 antibody (mouse monoclonal IgM antibody produced from a hybridoma (courtesy of Sommer et al.^70^) 1:100) for 1 h and fixed in 2% PFA for 7 min. Subsequently, OPCs were incubated with anti-SOX10 (R&D Systems AF2864 1:100) and anti-MBP (Abcam ab7349 1:200) primary antibodies for 1 h at RT, washed with 1× PBS, and incubated with secondary antibodies (anti-mouse IgM TRITC Southern Biotech 1021-03 1:100, donkey anti-goat Alexa 488 Abcam ab150129 1:1000, donkey anti-rat Alexa 647 Abcam 150155 1:750, and Hoechst H6024 Sigma 1:1000) for 1 h at RT, washed, and mounted with fluoromount. Images were obtained in an Axioscan (Zeiss).

### Statistical analyses

For qPCR, western blot, immunohistochemistry, electron microscopy, delayed alternation, set-shifting, and reversal learning data analysis, statistical significance was calculated using the independent samples *t*-test and, when appropriate, with Benjamini–Hochberg correction for multiple comparisons in IBM SPSS Statistics 24. Chi-square test was performed on axon caliber frequency data and a linear regression with two-way ANOVA was used to test statistical significance of the regression between G-ratio and axon caliber. For the environmental enrichment experiment, immunohistochemistry and electron microscopy data analysis were done using one-way ANOVAs, and effects were assessed with independent samples *t*-tests, and analysis of the set-shifting task was done using multivariate ANOVAs. For all analyses, outliers were discarded beforehand as indicated by the Grubbs outlier test using Graphpad quickcalcs 2018, and the level of significance was set at *p* = 0.05. All data were visualized using GraphPad Prism 6.01.

### Reporting summary

Further information on research design is available in the Nature Research Reporting Summary linked to this article.

## Supplementary information

Supplementary Information

Reporting Summary

## Data Availability

The datasets generated and analyzed during the current study are available from the corresponding author on reasonable request. The source data underlying Figs. 1–8 and Supplementary Figs. 1–5 are provided as a Source data file.

## Source Data

Source Data
