# Supplementary material for: Interneuron hypomyelination is associated with cognitive inflexibility in a rat model of schizophrenia
Source: Nat Commun. 2020 May 11;11:2329. doi: 10.1038/s41467-020-16218-4 (PMC7214427; doi:10.1038/s41467-020-16218-4)
Supplement: Supplementary file 1 — Supplementary Information [file 41467_2020_16218_MOESM1_ESM.pdf]

Supplementary information

Interneuron hypomyelination is associated with cognitive inflexibility in a rat model of schizophrenia

by Maas et al.

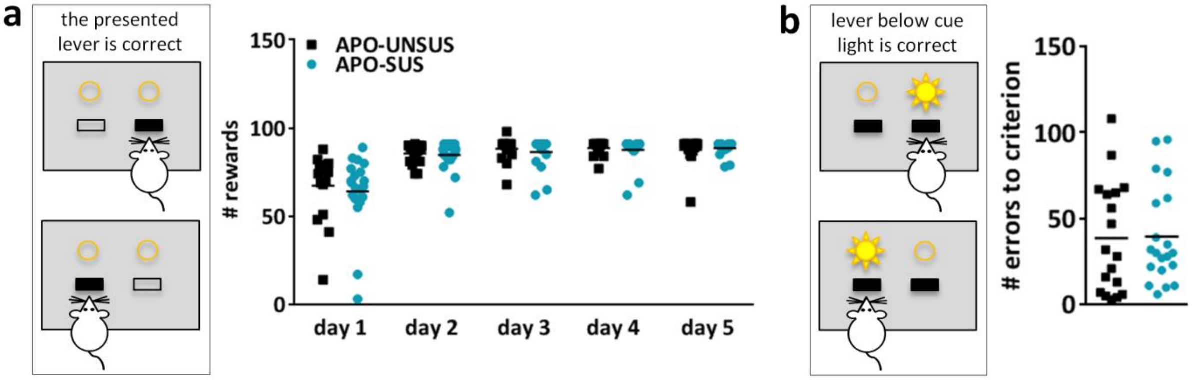

Supplementary Figure 1 – No difference in the performance of APO-SUS and APO-UNSUS rats during retractable lever-press training and visual cue discrimination.
(a) Schematic representation and performance of APO-SUS and APO-UNSUS rats during retractable lever press training. Rats were required to press the lever they were presented with during 5 consecutive sessions in 1 session per day. The number of rewards in each session in APO-SUS versus APO-UNSUS rats (n=18-20) is depicted. **(b)** Schematic representation of visual cue learning in the operant set-shifting paradigm. Rats were required to press the lever above which a cue light was illuminated in order to receive a reward pellet. The number of errors until criterion of a streak of 10 correct trials was reached in APO-SUS (n=20) and APO-UNSUS (n=18) rats is depicted. Source data are provided as a Source Data file.

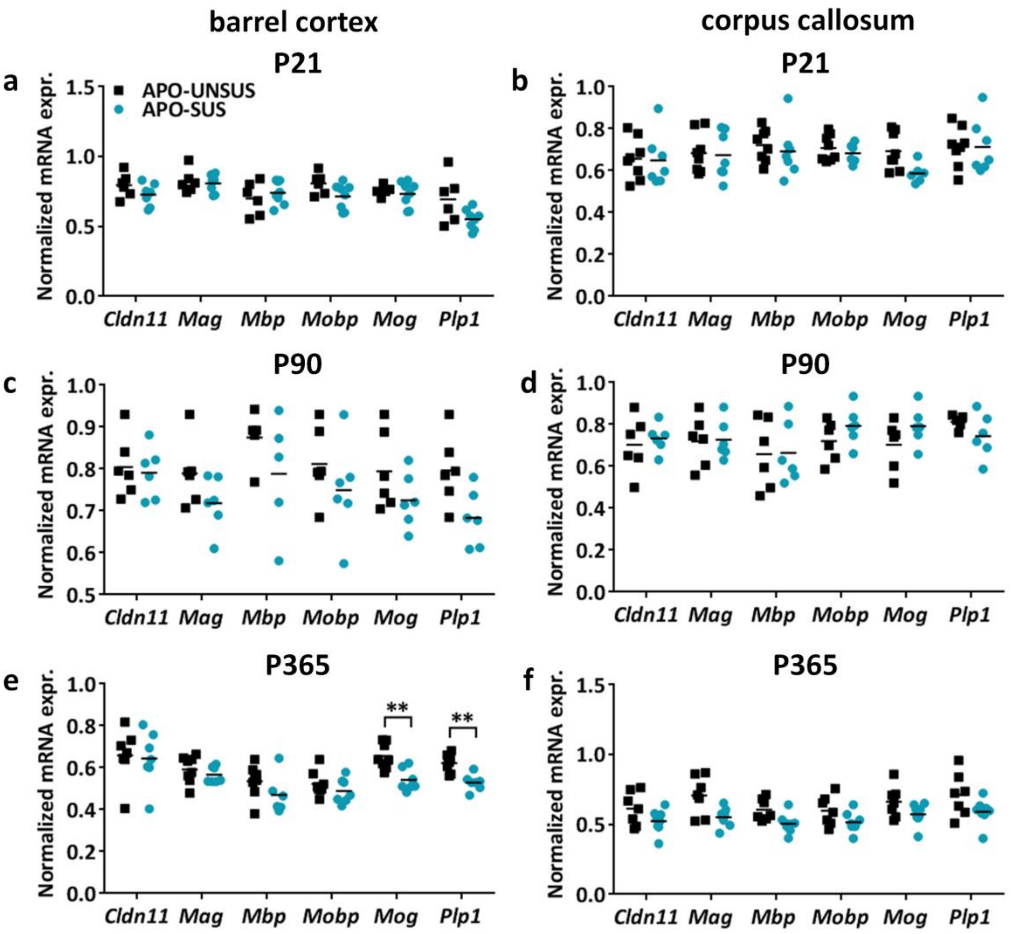

Supplementary Figure 2 – No difference in myelin-related mRNA expression in APO-SUS versus APO-UNSUS barrel cortex (BC) and corpus callosum (CC).

Normalized mRNA expression of the myelin-related genes proteolipid protein 1 (*Plp1*), myelin basic protein (*Mbp*), claudin 11 (*Cldn11*), myelin-associated oligodendrocytes basic protein (*Mobp*), myelin oligodendrocyte glycoprotein (*Mog*) and myelin associated glycoprotein (*Mag*) in BC and CC of P21 **(a-b),** P90 **(c-d)** and P365 **(e-f)** APO-SUS versus APO-UNSUS rats (P21 n=5-8; P90 n=5-6; P365 n=6-8). *Mog* P365 **p=0.002, *Plp1* P365 **p=0.001 in two-tailed independent samples T-test with Benjamini-Hochberg multiple comparisons correction. Source data are provided as a Source Data file.

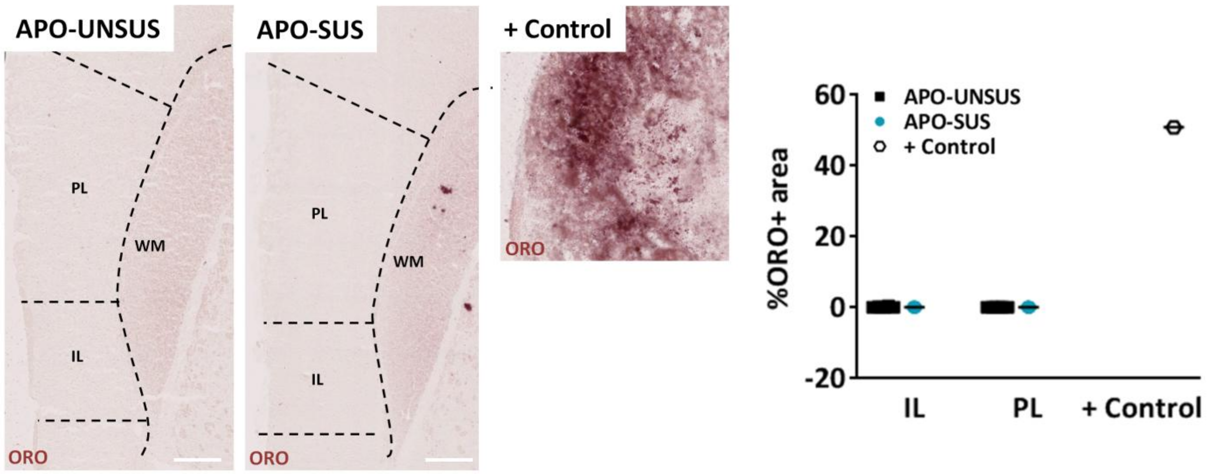

Supplementary Figure 3 – No active demyelination in APO-SUS and APO-UNSUS mPFC.
Immunohistochemical images and quantification of ORO staining, labeling myelin debris-containing macrophages, in APO-SUS (n=7) and APO-UNSUS (n=6) mPFC compared to a positive control (a demyelinating lesion induced by experimental autoimmune encephalomyelitis). Scale bars 200µm. Source data are provided as a Source Data file.

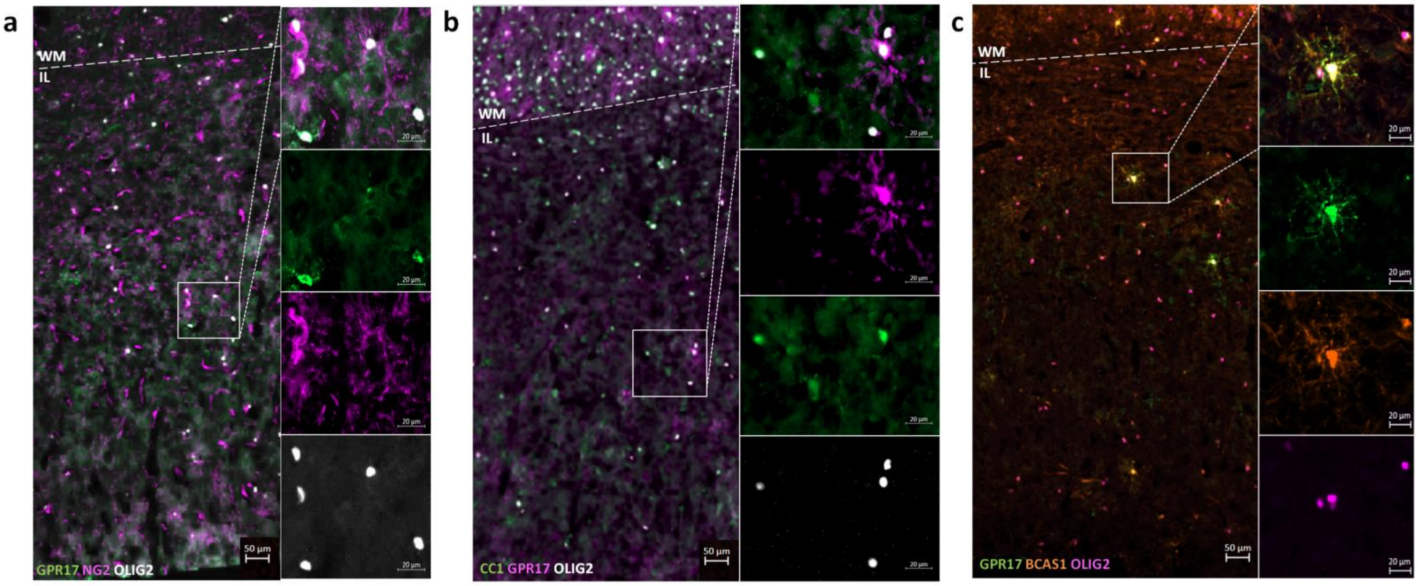

Supplementary Figure 4 – GPR17 marks immature OLs in the APO-SUS/APO-UNSUS mPFC and in combination with BCAS1 staining identifies early-myelinating OLs

(a) Coimmunostaining for GPR17, OLIG2 and NG2 reveals that the majority of GPR17+ OLs are not NG2+ OPCs. Staining performed in IL of APO-SUS (n=6) and APO-UNSUS (n=6) rats. **(b)** Coimmunostaining for GPR17, OLIG2 and CC1 shows that GPR17+ cells form a subset of CC1+ OLs. Staining performed in IL of APO-SUS (n=1) and APO-UNSUS (n=1) rats. **(c)** Coimmunostaining for GPR17, OLIG2 and BCAS1 shows that GPR17+ cells constitute a subset of newly formed BCAS1+ OLs. Scale bars (a, b, c) 50µm and 20µm in insets. Staining performed in IL of APO-SUS (n=13) and APO-UNSUS (n=12) rats.

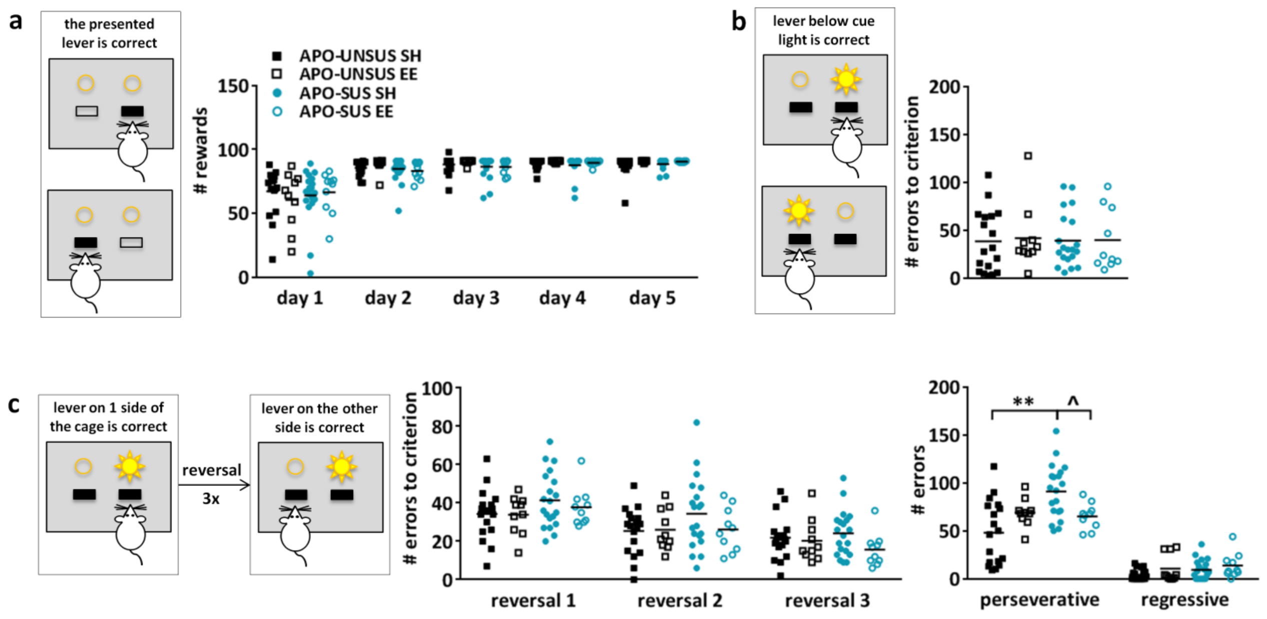

Supplementary Figure 5 – EE does not affect retractable lever press training or visual cue discrimination, but decreases the number of errors during reversal learning.
(a) Schematic representation and performance of APO-SUS and APO-UNSUS rats during retractable lever press training. Rats were required to press the lever they were presented with during 5 consecutive sessions in 1 session per day. The number of rewards in each session in APO-UNSUS and APO-SUS rats in SH (n=18-20) and EE (n=10) are depicted. **(b)** Schematic representation of visual cue discrimination in the operant set-shifting paradigm. Rats were required to press the lever above which a cue light was illuminated in order to receive a reward. The number of errors until criterion of a streak of 10 correct trials was reached in APO-UNSUS and APO-SUS rats in SH (n=18-20) and EE (n=10) are depicted. Error bars represent standard error of the mean. **(c)** Schematic representation of operant reversal learning paradigm. Rats were trained to press the lever on one side of the cage until criterion of a streak of 10 correct trials was reached. In the following session from trial 21 onwards rats were required to press the lever on the other side of the operant cage. Number of errors until criterion of a streak of 10 correct trials, and total number of perseverative and regressive errors in APO-UNSUS and APO-SUS rats in SH (n=18-20) and EE (n=10) are depicted. **p<0.0001 APO-SUS SH versus APO-UNSUS SH ^p=0.014 APO-SUS SH versus EE in two-tailed multivariate ANOVA post hoc testing. Source data are provided as a Source Data file.

Supplementary Table 1 – Names and nucleotide sequences of primers used for qPCRs.

Gene and abbreviation		Forward primer 5'-3'	Reverse primer 5'-3'
**Housekeeping genes**			
β-actin	Beta-actin	CCTTCCTGGGTATGGAATCCTGT	TAGAGCCACCAATCCACACA
Ppia	Peptidyl-prolyl cis-trans isomerase A	AGCACTGGGGAGAAAGGATT	AGCCACTCAGTCTTGGCAGT
Gapdh	Glyceraldehyde-3-phosphate	GGGTGTGAACCACGAGAAAT	ACTGTGGTCATGAGCCCTTC
Ywhaz	Tyrosine 3-monooxygenase/tryptophan 5-monooxygenase activation protein, zeta	TTGAGCAGAAGACGGAAGGT	GAAGCATTGGGGATCAAGAA
**Myelin-related genes**			
Cldn11	Claudin 11	CGCAAAATGGACGAACTGGG	TGCACGTAACCAGGGAGGAT
Mag	Myelin-associated glycoprotein	AAGCCAGACCATCCAACCTTC	CTCCTGATTCCGCTCCAAGT
Mbp	Myelin basic protein	CCCTACTCCATCCTCAGACTTTC TT	TGGCGGTGTGCCTGTCTAT
Mobp	Myelin-associated oligodendrocytebasic protein	AATACCTGCAGGGCAACAAAG	TCTGGTTCTTGGAGAGCCTGG
Mog	Myelin oligodendrocyte glycoprotein	CGCCGTGGAGTTGAAAGTAG	GCACGGAGTTTTCCTCTAGT
Plp1	Proteolipid protein 1	GGGCCTGAGCGCAACGGTAA	CAGGCACAGCAGAGCAGGCAA

Supplementary Table 2 – Statistical values of qPCR timeline determined by two-tailed independent samples T-test with Benjamini-Hochberg multiple comparisons correction.

Brain region	Gene	Age	t-value	p-value	Sample size APO-SUS	Sample size APO-UNSUS	Significant after Benjamini Hochberg correction
**PFC**	***Plp1***	**P0**	-1.248	0.230	10	8	No
		**P7**	0.804	0.432	8	12	No
		**P14**	-0.432	0.670	12	11	No
		**P21**	-3.256	0.006	7	8	Yes
		**P28**	-3.915	0.004	8	8	Yes
		**P90**	-3.749	0.006	5	5	Yes
		**P365**	-3.419	0.008	7	8	Yes
	Mbp	**P0**	0.067	0.948	10	8	No
		**P7**	0.941	0.359	8	12	No
		**P14**	0.101	0.921	12	12	No
		**P21**	-0.568	0.576	10	12	No
		**P28**	-5.150	0.001	8	8	Yes
		**P90**	-6.684	<0.0001	4	5	Yes
		**P365**	-5.097	0.001	8	8	Yes
	Cldn11	**P0**	-1.705	0.125	10	8	No
		**P7**	-0.432	0.670	8	12	No
		**P14**	0.378	0.709	12	11	No
		**P21**	-2.149	0.051	7	8	No
		**P28**	-4.313	0.001	8	7	Yes
		**P90**	-7.245	<0.0001	4	6	Yes
		**P365**	-0.495	0.628	8	8	No
	Mobp	**P21**	-3.431	0.011	6	7	Yes

		**P28**	-5.848	0.001	8	7	Yes
		**P90**	-4.751	0.002	4	6	Yes
		**P365**	-3.954	0.002	8	8	Yes
	Mog	**P21**	-2.493	0.038	6	8	No
		**P28**	-4.736	0.001	8	8	Yes
		**P90**	-17.440	<0.0001	4	5	Yes
		**P365**	-2.263	0.040	8	8	Yes
	Mag	**P21**	-5.526	<0.0001	6	8	Yes
		**P28**	-7.122	<0.0001	6	8	Yes
		**P90**	-3.132	0.012	5	6	Yes
		**P365**	-3.393	0.010	7	8	Yes
**BC**	***Plp1***	**P21**	-1.946	0.097	8	5	No
		**P90**	-2.581	0.027	6	6	No
		**P365**	-4.447	0.001	7	8	Yes
	Mbp	**P21**	0.751	0.467	8	6	No
		**P90**	-1.249	0.247	5	5	No
		**P365**	-1.431	0.178	6	8	No
	Cldn11	**P21**	-1.722	0.113	7	6	No
		**P90**	-0.360	0.726	6	6	No
		**P365**	-0.217	0.832	7	7	No
	Mobp	**P21**	-2.032	0.065	8	6	No
		**P90**	-1.068	0.311	6	6	No
		**P365**	-1.108	0.288	7	8	No
	Mog	**P21**	-0.625	0.546	8	5	No
		**P90**	-1.490	0.167	6	6	No

		**P365**	-2.844	0.002	7	8	Yes
	Mag	**P21**	-0.375	0.714	8	6	No
		**P90**	-1.718	0.117	6	6	No
		**P365**	-0.936	0.366	7	8	No
**CC**	***Plp1***	**P21**	-0.005	0.996	7	8	No
		**P90**	-1.363	0.206	6	5	No
		**P365**	-1.900	0.080	8	7	No
	Mbp	**P21**	-0.561	0.584	7	8	No
		**P90**	0.065	0.949	6	6	No
		**P365**	-2.669	0.019	8	7	No
	Cldn11	**P21**	-0.143	0.889	7	8	No
		**P90**	0.496	0.630	6	6	No
		**P365**	-1.653	0.128	8	7	No
	Mobp	**P21**	-0.842	0.416	6	8	No
		**P90**	1.337	0.211	6	6	No
		**P365**	-1.803	0.095	8	7	No
	Mog	**P21**	-2.910	0.014	6	8	No
		**P90**	1.461	0.175	6	6	No
		**P365**	-1.819	0.092	8	7	No
	Mag	**P21**	-0.182	0.858	7	8	No
		**P90**	0.114	0.912	6	6	No
		P365	-2.776	0.016	8	7	No
