# Supplementary material for: Interneuron hypomyelination is associated with cognitive inflexibility in a rat model of schizophrenia
Source: Nat Commun. 2020 May 11;11:2329. doi: 10.1038/s41467-020-16218-4 (PMC7214427; doi:10.1038/s41467-020-16218-4)
Supplement: Supplementary file 2 — Reporting Summary [file 41467_2020_16218_MOESM2_ESM.pdf]

nature research

Corresponding author(s): Dorien A. Maas

Last updated by author(s): Apr 6, 2020

Reporting Summary

Nature Research wishes to improve the reproducibility of the work that we publish. This form provides structure for consistency and transparency in reporting. For further information on Nature Research policies, see Authors & Referees and the Editorial Policy Checklist .

Statistics

For all statistical analyses, confirm that the following items are present in the figure legend, table legend, main text, or Methods section.

n/a	Confirmed	
☐	☒	The exact sample size (n) for each experimental group/condition, given as a discrete number and unit of measurement
☐	☒	A statement on whether measurements were taken from distinct samples or whether the same sample was measured repeatedly
☐	☒	The statistical test(s) used AND whether they are one- or two-sided<br>*Only common tests should be described solely by name; describe more complex techniques in the Methods section.*
☐	☒	A description of all covariates tested
☐	☒	A description of any assumptions or corrections, such as tests of normality and adjustment for multiple comparisons
☐	☒	A full description of the statistical parameters including central tendency (e.g. means) or other basic estimates (e.g. regression coefficient) AND variation (e.g. standard deviation) or associated estimates of uncertainty (e.g. confidence intervals)
☐	☒	For null hypothesis testing, the test statistic (e.g. F, t, r) with confidence intervals, effect sizes, degrees of freedom and P value noted<br>*Give P values as exact values whenever suitable.*
☒	☐	For Bayesian analysis, information on the choice of priors and Markov chain Monte Carlo settings
☒	☐	For hierarchical and complex designs, identification of the appropriate level for tests and full reporting of outcomes
☒	☐	Estimates of effect sizes (e.g. Cohen's d, Pearson's r), indicating how they were calculated

Our web collection on statistics for biologists contains articles on many of the points above.

Software and code

Policy information about availability of computer code

Data collection	Image acquisition: Apotome (Leica), Axiocan (Zeiss), Zeiss Zen Blue Edition 2.3, Hitachi HT7700, and ImageQuant LAS4000<br>Operant behavioral testing: MED-PC V<br>qPCR: sample pipetting with Corbett robotics 4 and data acquisition with Rotor-Gene Q Series 2.0.2.
Data analysis	Image analysis: Zen Blue lite 2.3 or Fiji-ImageJ 1.51<br>qPCR data normalisation: GeNorm 39<br>Statistics: IBM SPSS Statistics 24 and GraphPad Quickcalcs 2018<br>Data visualisation: GraphPad Prism 6.01<br>Primer design: Primer Express 2.0.

For manuscripts utilizing custom algorithms or software that are central to the research but not yet described in published literature, software must be made available to editors/reviewers. We strongly encourage code deposition in a community repository (e.g. GitHub). See the Nature Research guidelines for submitting code & software for further information.

Data

Policy information about availability of data

All manuscripts must include a data availability statement. This statement should provide the following information, where applicable:

- Accession codes, unique identifiers, or web links for publicly available datasets
- A list of figures that have associated raw data
- A description of any restrictions on data availability

The datasets generated and analysed during the current study are available from the corresponding author on reasonable request. The source data underlying Figures 1-8 and Supplementary Figures 1-5 are provided as a Source Data file.

Field-specific reporting

Please select the one below that is the best fit for your research. If you are not sure, read the appropriate sections before making your selection.

[x] Life sciences [ ] Behavioural & social sciences [ ] Ecological, evolutionary & environmental sciences

For a reference copy of the document with all sections, see nature.com/documents/nr-reporting-summary-flat.pdf

Life sciences study design

All studies must disclose on these points even when the disclosure is negative.

Sample size	Sample size approximation for western blot and immunofluorescence experiments were based on and in line with the APO-SUS and APO-UNSUS rat experiments of Selten et al., Sci Rep, 6:34240 (2016). Sample size approximations for behavioral experiments and qPCR experiments were based on and in line with the APO-SUS and APO-UNSUS rat experiments of Coolen et al., Neuron 45:497 (2005). Electron microscopy experiments and immunofluorescence on ultrathin sections were informed by unpublished pilot study data, which reflect the outcomes of the experiments performed in this study
Data exclusions	Data were only excluded from analysis if they were statistically significant outliers as determined by Grubbs' outlier test performed in Graphpad Quickcalcs. This led to the exclusion of one datapoint from the 60 sec delay in the delayed alternation experiment (Fig 1a), from the qPCR experiment (Fig 2a), from the mPFC western blot experiment (Fig 2b), from the ultrathin immunofluorescence experiment (Fig 4a), from the immunohistochemistry experiments (Fig 5d and 7e).
Replication	Part of our immunohistochemical experiments (OL lineage cell, OL precursor cell and mature OL stainings) have been independently replicated once in brain tissue from a second batch of animals. These replications were successful. Our qPCR analyses have been successfully and independently replicated three times in samples from animals of 60, 120 and 180 days old. All behavioral experiments on control APO-SUS and APO-UNSUS rats have been successfully replicated in a second experiment performed by another, independent researcher.
Randomization	Animals from the same litter were randomly allocated to different experimental groups to avoid litter bias.
Blinding	Blinding was applied during all experiments and all experimental stages, except for housing conditions during the environmental enrichment paradigm, which cannot be blinded for practical reasons. During the behavioral experiments all animals were given a number and after the analysis was revealed to which experimental group the animal numbers corresponded.

Reporting for specific materials, systems and methods

We require information from authors about some types of materials, experimental systems and methods used in many studies. Here, indicate whether each material, system or method listed is relevant to your study. If you are not sure if a list item applies to your research, read the appropriate section before selecting a response.

Materials & experimental systems

n/a	Involved in the study	
[ ]	[x]	Antibodies
[x]	[ ]	Eukaryotic cell lines
[x]	[ ]	Palaeontology
[ ]	[x]	Animals and other organisms
[x]	[ ]	Human research participants
[x]	[ ]	Clinical data

Methods

n/a	Involved in the study	
[x]	[ ]	ChIP-seq
[x]	[ ]	Flow cytometry
[x]	[ ]	MRI-based neuroimaging

Antibodies

Antibodies used

Primary antibodies:
- anti-OLIG2: Millipore cat. number AB9610 and MABN50, Abcam cat. number ab109186
- Anti-APC (Ab-7, (clone CC-1): Calbiochem cat. number OP80
- anti-NG2: Millipore cat. number MAB5384 and AB5320
- anti-PLP: Biorad cat. number MCA839G
- Hoechst: Sigma cat. number H6024
- anti-MBP: Aves cat. number MBP, Abcam cat. number ab7349, and Millipore cat. number MAB386
- anti-GABA: Millipore, cat. number AB175
- anti-O4: mouse monoclonal IgM antibody produced from a hybridoma (courtesy of Sommer and Schachner, Dev Biol 83:311-327 (1981))
- anti-SOX10: R&D Systems cat.number AF2864
- anti-GAPDH: Cell Signaling cat. number 2118

- anti-GPR17: Cayman Chemical cat. number 10136
- anti-PV: Swant cat. number PV27
-anti-BCAS1: Santa Cruz cat. number sc-136342

Secondary antibodies:
- Alexa 488-conjugated goat anti-rabbit IgG: Abcam cat. number ab150077
- Alexa 555-conjuagted goat anti-rabbit IgG: Abcam cat. number ab150078
- Alexa 568-conjugated goat anti-mouse IgG2b: Thermo Fisher cat. number A-21144
- TRITC-conjugated goat anti-mouse IgG1: Southern Biotech cat. number ABIN376752
- Alexa 647-conjugated goat anti-mouse IgG2a: Jackson Immuno Research cat. number 115-605-206
- TRITC-conjuagted donkey anti-mouse IgM: Southern Biotech cat. number 1021-03
- Alexa 488-conjugated donkey anti-goat IgG: Abcam cat. number ab150129
- Alexa 647-conjuagted donkey anti-rat IgG: Abcam cat. number ab150155
- Alexa 488-conjuagted goat anti-guinea pig IgG: Thermo Fisher cat. number A-11073
- Alexa 594-conjuagted goat anti-chicken IgY: Thermo Fisher cat. number A-11042
- HRP-conjugated goat anti-rabbit IgG: Nordic Immunology cat. number: GAR/IgG(H+L)/PO
- HRP-conjugated anti-mouse IgG: Nordic Immunology cat. number: GAM/IgG(H+L)/PO

Validation

Primary antibodies were validated as follows:
- anti-OLIG2: Millipore cat. number AB9610: validated by the manufacturer "Evaluated by immunohistochemistry on glioblastoma" and "Human, rat and mouse. Other species have not been tested"
-anti-OLIG2: Millipore cat. number MABN50: validated by the manufacturer "Detect Olig2 using this Anti-Olig2 Antibody, clone 211F1.1 validated for use in WB, IC, IH & IP" and "Demonstrated to react with mouse. Predicted to react with human and rat based on 100% sequence homology"
-anti-OLIG2: Abcam cat. number ab109186: validated by the manufacturer "Our Abpromise guarantee covers the use of ab42453 in the following tested applications... IHC (PFA fixed) 1/1000"
- anti-CAPC (CC1): Calbiochem cat. number OP80: validated by the manufacturer "Well suited for IHC and IF studies of oligodendrocytes and optic nerves due to the antibody's staining of the cell body as opposed to the myelinated processes. Both frozen sections and formalin-fixed, paraffin-embedded brain sections can be used. Antibody should be titrated for optimal results in individual systems" and "species reactivity: Human Mouse Rat"
- anti-NG2: Millipore cat. number MAB5384: validated by the manufacturer ".
- Anti-NG2 , clone 132.38, Cat. No. MAB5384-I, is a highly specific mouse monoclonal antibody that targets Chondroitiin Sulfate Protoglycan 4 and has been tested for use in Immunofluorescence and Western Blotting" and "species reactivity: Rat Mouse".
- anti-NG2: Millipore cat. number AB5320: validated by the manufacturer "Detect NG2 Chondroitin Sulfate Proteoglycan using this Anti-NG2 Chondroitin Sulfate Proteoglycan. Antibody validated for use in ELISA, IC, IH, IP and WB" and "species reactivity: Human Mouse Rat Monkey"
- anti-PLP: Biorad cat. number MCA839G: validated by the manufacturer ". This product has been reported to work in the following applications. This information is derived from testing within our laboratories, peer-reviewed publications or personal communications from the originators ... Immunohistology – Frozen".
- anti-MBP: Aves cat. number MBP: validated by the manufacturer. "Species Reactivity: Human, Mouse, Rat" and "Applications: ICC, IHC, WB" and "Immunohistochemical staining of the MBP.
-anti-MBP: Abcam cat. number ab7349: validated by the manufacturer "Our Abpromise guarantee covers the use of ab7349 in the following tested applications ... ICC/IF: Use at an assay dependent concentration. PubMed: 23584610"
-anti-MBP: Millipore cat. number MAB386: validated by the manufacturer "Western Blotting:
Identifies an 18-20kDa band in westerns"and "Reacts with MBP from all species tested including human, bovine, sheep, rabbit, mouse, rat, guinea pig and chicken".
- anti-GABA: Millipore, cat. number AB175: validated by the manufacturer "Immunohistochemistry Analysis: A 1:500 dilution from a representative lot detected GABA in rat brain tissue".
- anti-O4: Home made antibody from a hybridoma purchased from ATCC. This antibody has been validated extensively in OL cell lineage cell cultures, as well as mouse and rat brain and spinal cord tissue.
- anti-SOX10: R&D Systems cat. number AF2864: validated by the manufacturer "SOX10 was detected in immersion fixed BG01V human embryonic stem cells differentiated to neural crest stem cells using Goat Anti-Human SOX10 Antigen Affinity-purified Polyclonal Antibody (Catalog # AF2864) at 10 µg/mL for 3 hours at room temperature".
- anti-GAPDH: Cell Signaling cat. number 2118: validated by the manufacturer "Supporting data: Specificity / Sensitivity: GAPDH (14C10). Rabbit mAb detects endogenous levels of total GAPDH protein. Species Reactivity: Human, Mouse, Rat, Monkey, Bovine, Pig"
- anti GPR17: Cayman Chemical cat. number 10136: validated by the manufacturer "Applications: IHC and WB" and "Species Reactivity (+) Human GPR17 (+) Mouse GPR17 (+) Rat GPR17".
- anti-PV: Swant cat. number PV27: validated by the manufacturer . "This antiserum was produced against recombinant rat parvalbumin. It cross-reacts with many other species, humans included. It can be used in immunohistochemistry and for immunoblotting".
-anti-BCAS1: Santa Cruz cat. number sc-136342: validated by the manufacturer "NaBC1 (5) is recommended for detection of NaBC1 of human origin by Western Blotting (starting dilution 1:200, dilution range 1:100-1:1000), immunoprecipitation [1-2 µg per 100-500 µg of total protein (1 ml of celllysate)] and immunofluorescence (starting dilution 1:50, dilution range1:50-1:500".

Animals and other organisms

Policy information about studies involving animals; ARRIVE guidelines recommended for reporting animal research

Laboratory animals	APO-SUS and APO-UNSUS rat lines were used in this study. APO-SUS and APO-UNSUS rats have been selectively bred from an outbred Nijmegen Wistar rat population based on stereotyped behavior upon injection of apomorphine. Apomorphine injection and behavioral selection were only performed with the first 15 generations of APO-SUS and APO-UNSUS rats. In the subsequent breedings and in the breedings used in this study, APO-SUS rats did not receive any pharmacological treatment. In this study naïve male APO-SUS and APO-UNSUS rats from the 38th-43rd generation were used. Behavioral experiments and environmental enrichment were conducted with adult 60-90 days old rats. For qPCR experiments rats of 0, 7, 14, 21, 28, 90 and 365 days old were used. For all other experiments 90 days old rats were used.
Wild animals	The study did not involve wild animals.
Field-collected samples	The study did not include field-collected samples.
Ethics oversight	Animal experiments were approved by the Animal Ethics Committee of Radboud University Nijmegen Medical Centre, Nijmegen, the Netherlands, and were conducted in accordance with Dutch legislation (Herziene Wet op Dierproeven, Art 10.a.2, 2014).

Note that full information on the approval of the study protocol must also be provided in the manuscript.
